# Six new species and reports of *Hydnum* (Cantharellales) from eastern North America

**DOI:** 10.3897/mycokeys.42.27369

**Published:** 2018-11-30

**Authors:** Rachel A. Swenie, Timothy J. Baroni, P. Brandon Matheny

**Affiliations:** 1 Department of Ecology and Evolutionary Biology, 569 Dabney Hall, University of Tennessee, Knoxville, TN 37996, USA University of Tennessee Knoxville United States of America; 2 Department of Biological Sciences, PO Box 2000, State University of New York, College at Cortland, Cortland, New York 13045, USA State University of New York New York United States of America

**Keywords:** Basidiomycota, Agaricomycetes, Hydnaceae, ectomycorrhizal fungi, taxonomy, systematics, type studies

## Abstract

Five species of *Hydnum* have been generally recognized from eastern North America based on morphological recognition: *H.albidum*, *H.albomagnum*, *H.repandum* and varieties, *H.rufescens*, and *H.umbilicatum*. Other unique North American species, such as *H.caespitosum* and *H.washingtonianum*, are either illegitimately named or considered synonymous with European taxa. Here, seventeen phylogenetic species of *Hydnum* are detected from eastern North America based on a molecular phylogenetic survey of ITS sequences from herbarium collections and GenBank data, including environmental sequences. Based on current distribution results, sixteen of these species appear endemic to North America. Of these, six species are described as new: *H.alboaurantiacum*, *H.cuspidatum*, *H.ferruginescens*, *H.subconnatum*, *H.subtilior*, and *H.vagabundum*. Geographic range extensions and taxonomic notes are provided for five additional species recently described as new from eastern North America. A new name, *H.geminum*, is proposed for *H.caespitosum* Banning ex Peck, non Valenti. Overall, species of *Hydnum* are best recognized by a combination of morphological and molecular phylogenetic analyses. Taxonomic descriptions are provided for seventeen species, including epitype designations for *H.albidum*, *H.albomagnum*, and *H.umbilicatum*, taxa described more than 100 years ago, and molecular annotation of the isotype of *H.washingtonianum*. Photographs and a key to eastern North American *Hydnum* species are presented.

## Introduction

*Hydnum* L: Fr (=*Dentinum* Gray) is a genus of ectomycorrhizal (ECM) mushroom-forming fungi found primarily in temperate forests of Asia, Australia, Europe, and North America. Until recently, only twelve species of *Hydnum* were commonly accepted worldwide. Initial phylogenetic studies of European *Hydnum* revealed higher than expected taxonomic diversity in the genus, with thirteen molecularly recognized clades masquerading under four morphologically-defined species names (Grebenc et al. 2009), which have since been described as new species (Olariaga et al. 2012, Vizzini et al. 2013, Niskanen et al. 2018). Following a global survey of diversity in the genus which estimated 31 species worldwide based on molecular phylogenetic analysis (Feng et al. 2016), additional taxonomic work in Europe and North America has raised the global species count to 34 (Buyck et al. 2017, Niskanen et al. 2018), which is estimated to be less than half of the total number of *Hydnum* species (Niskanen et al. 2018). This previously overlooked diversity may be due to morphological stasis in evolution of basidiome morphology and lack of attention to regional characterization of the *Hydnum* flora in locations outside Europe.

*Hydnum*, in its original Linnean concept, contained all species of mushroom-forming fungi with a spinose hymenophore (e.g., Fries 1821). Indeed, over 900 names have been attributed to the genus per Index Fungorum (www.indexfungorum.org). However, molecular phylogenetic analysis showed that the spinose hymenophore has evolved independently many times in distantly related taxa (Hibbett et al. 1997). As a consequence, most species formerly contained in *Hydnum* have been moved to other genera. Species of *Hydnum* in the contemporary sense (Donk 1956) and typified by *H.repandum* L.:Fr. are united by their smooth hyaline basidiospores, white to orange basidiomes, and stichic basidia, in which the meiotic spindle is vertically oriented (Donk 1933, Maas Geesteranus 1971, Restivo and Petersen 1976, Pine et al. 1999). Ecologically, *Hydnum* form ECM associations with a variety of vascular plant species including members of Pinaceae (Agerer et. al 1996), Myrtaceae (McNabb 1971), Fagales (McNabb 1971, Feng et. al 2016, Niskanen et al. 2018), Salicaceae (Niskanen et al. 2018), Malvaceae (Niskanen et al. 2018), and Dipterocarpaceae (Lee et al. 2002). The genus is distributed mostly in temperate areas, with a few reports from tropical and subtropical forests in southeast Asia (Lee et al. 2002, Feng et al. 2016) and the neotropics (Garibay-Orijel et al. 2006, Sarmiento and Fontecha 2013, Feng et al. 2016, Niskanen et al. 2018).

Previous analysis of global *Hydnum* diversity revealed the presence of six distinct clades of *Hydnum* in eastern North America, only one of which also occurs on another continent (Feng et al. 2016). Currently, five species have been described from eastern North America: *H.albidum* Peck, *H.albomagnum* Banker, *H.aerostatisporum* Buyck, D.P. Lewis & V. Hofst., *H.caespitosum* Banning ex Peck (*non* Valenti), and *H.umbilicatum* Peck. Banker (1906) and Harrison and Grund (1987) recognized six species of *Hydnum* from across North America in their taxonomic treatments. Of those species that occur in eastern North America, four were described over one hundred years ago, and the application of those names has not been clarified in light of molecular phylogenetic analyses. Here, we resolve those species names by taxonomic revision of type specimens and DNA sequencing of contemporary collections, as well as document *Hydnum* species diversity and distribution in eastern North America. Seventeen species are treated here, of which six are described as new. A taxonomic key to species from eastern North America is included.

## Methods

Dried specimens of *Hydnum* were obtained from TENN, CORT, NYS, NY, WTU, and CSU. Herbarium abbreviations follow Thiers [continuously updated]. Additional collections were borrowed from the personal herbarium of Michael Kuo (Charleston, Illinois). Fresh *Hydnum* specimens were collected from localities in the eastern United States (North Carolina, Tennessee, Georgia, Florida, Virginia). Color documentation of fresh material follows Kornerup and Wanscher (1967; e.g., 5A2), Munsell Soil Color Charts (1954; e.g., 10YR 4/7), or Ridgway (1912; e.g., “Ochraceous-Tawny”). Macroscopic descriptions were taken from fresh material. In some instances, 5% KOH and 10% FeSO_4_ were applied to pilei to test for macrochemical reactions.

Microscopic features were examined on a Nikon Eclipse 80i microscope from dried material rehydrated in 5% KOH and stained with Congo red or phloxine. Measurements and photographs were taken using a Nikon DS-Fi1 camera and Nikon NIS Elements 3.1 software. Basidiospores were measured from spore prints where available or spine tissue, and *Q* (quotient of basidiospore length to width) was calculated for each spore. The number of spores measured for each species is represented as n=total number/number of specimens (e.g., n=20/3). Measurements in excess of two standard deviations are denoted in parentheses and averages in italics.

DNA extractions were performed using two methods. Fresh or dried material less than five years old was extracted using an Extract-N-Amp Plant kit (Sigma-Aldrich, St. Louis, MO, USA). Older dried specimens were extracted using an HP Fungal DNA Extraction Kit (Omega Bio-Tek, Norcross, Georgia, USA). For specimens >50 years old, 10–20 mg ground tissue was incubated in extraction buffer at 65 °C for 72 hours prior to the first extraction step. The extraction was performed in a laminar flow hood to minimize contamination.

Primers ITS1F and ITS4 (Gardes and Bruns 1993, White et al. 1990) were used to amplify and sequence the nuclear rDNA internal transcribed spacer 1, 5.8S rRNA, and internal transcribed spacer 2 (hereafter, ITS). For older materials, we amplified and sequenced the two spacer regions separately following Ammirati et al. (2007) using primers ITS1F/ITS2 and 5.8SR/ITS4. Sequencing was performed on an Applied Biosystems 3730 Genetic Analyzer at the University of Tennessee Genomics Core. Sequence reads were assembled using Sequencher 5.0.1 (Gene Codes Corp., Ann Arbor, Michigan, USA).

GenBank sequences labeled as *Hydnum*, as well as closely matching environmental sequences, were downloaded. Sequences were visualized in AliView 1.20 (Larsson 2014) and aligned using MUSCLE 3.8.31 (Edgar 2004). Minor adjustments were made manually to the alignment. The GTR+I+GAMMA substitution model was selected as the best-fit model for Bayesian inference (BI) analysis in PartitionFinder 2.1.1 (Lanfear et al. 2016). BI analyses were performed using MrBayes 3.2.6 (Ronquist and Huelsenbeck 2003); the global analysis ran for 10 million generations with sampling every 1000^th^ generation. Following global BI tree visualization, clades that did not contain sequences from eastern North America were pruned to form a second tree figure for easier graphical representation. Maximum Likelihood (ML) analysis was performed on the pruned dataset in RAxML 8.2.8 (Stamatakis 2014) using 1000 bootstraps under a GTRGAMMA model of nucleotide substitution following the RAxML user manual recommendation. BI analysis of the pruned data set ran for 5 million generations with sampling every 500^th^ generation.

The resulting phylogenies were visualized in FigTree v.1.4.3 (http://tree.bio.ed.ac.uk/software/figtree/). Mislabeled sequences were omitted, as well as *Hydnum* from the southern hemisphere due to high levels of sequence divergence (Feng et al. 2016). *Sistotremaalboluteum* was chosen as an outgroup based on Feng et al. (2016). DNA alignments and tree files are available at TreeBase (submission 22888).

Species are recognized here as monophyletic groups that differ in morphology, ecology, and/or geographic distribution.

## Results

119 ITS sequences were produced for this study (Suppl. material 1). For the BI analyses, the average standard deviation of split frequencies reached below 0.01 after 4 million generations (global phylogeny) and 2 million generations (pruned phylogeny), so the first 40% of sampled trees were discarded as the burn-in for each analysis. Posterior probabilities (PP) for each analysis were calculated from 12002 samples from two independent runs for both the global and pruned analyses. In each case the potential scale reduction factor (PSRF) convergence diagnostic reached a value of 1.0 for all parameters, indicative of sufficient sample size.

**Figure 1.**
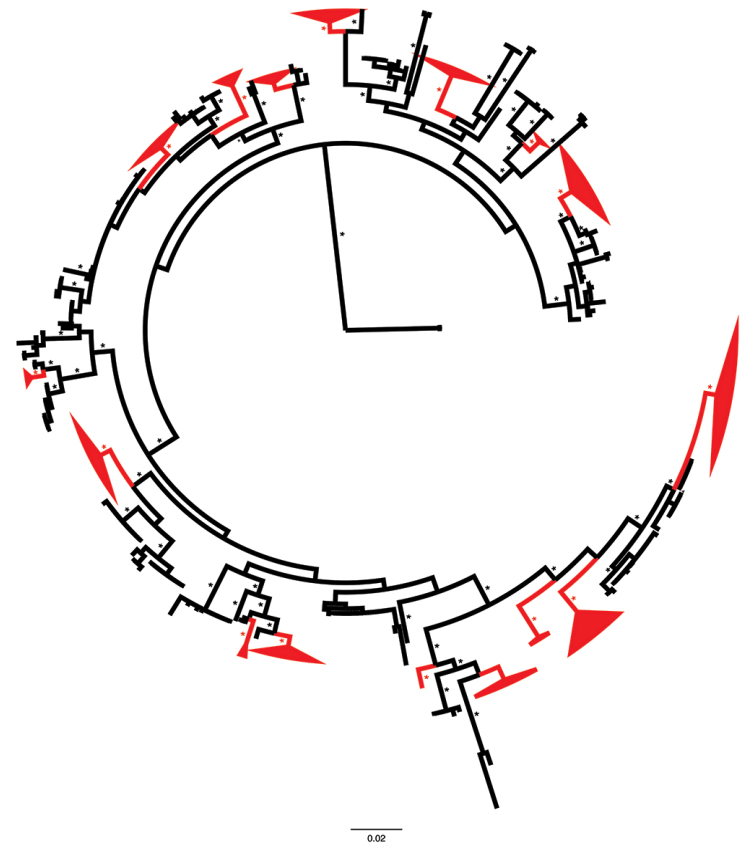
Global BI phylogeny of *Hydnum*. Species-level clades containing sequences from eastern United States and Canada are colored red. Posterior probabilities ≥ 0.95 are denoted with asterisks.

**Figure 2.**
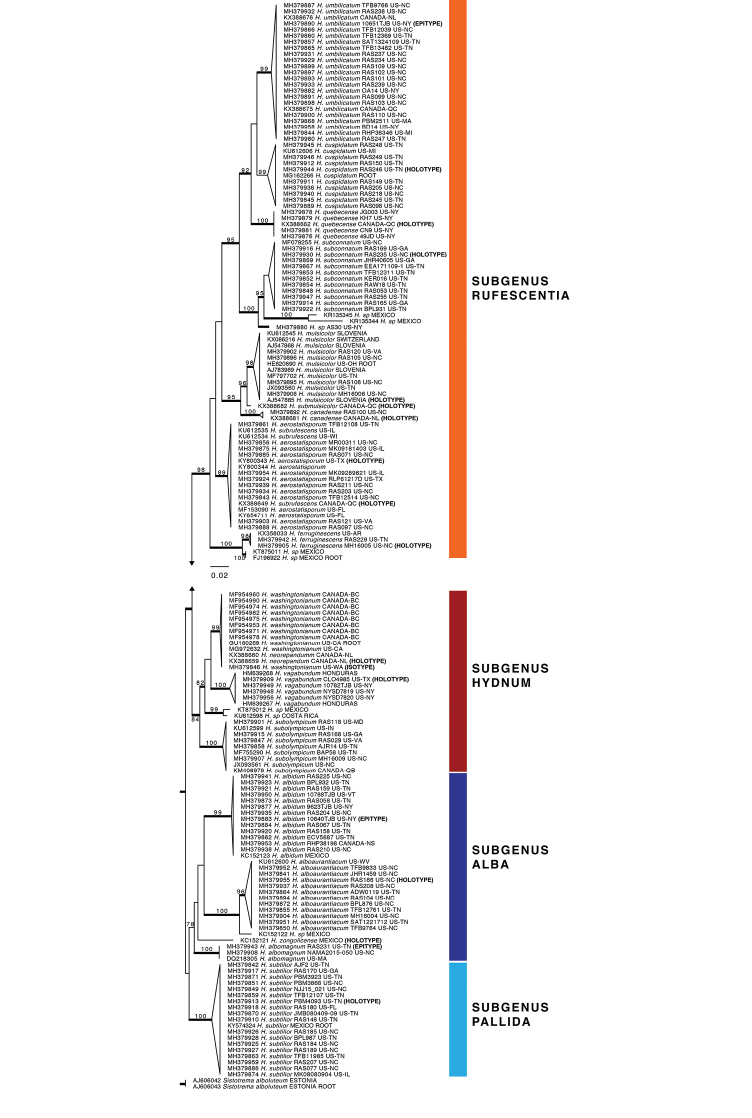
ML pruned phylogeny of North American species of *Hydnum*. Bootstrap support values ≥70% are shown above branches. Posterior probabilities ≥0.95 are shown with branches in bold.

The global phylogeny contained 397 sequences and 61 species-level clades (Suppl. material 2). Species-level clades containing sequences from eastern United States and Canada are colored red in the global circle tree (Fig. 1). PP ≥ 0.95 for nodes of these clades are denoted with an asterisk. The pruned version of the global tree, including taxon tip labeling information, is shown in Fig. 2. Four major clades of North American *Hydnum* were recovered, each with ML bootstrap support ≥ 70%. These are labeled by subgenus: *Alba*, *Pallida*, *Hydnum*, *Rufescentia* (Niskanen et al. 2018). A sister group relationship was recovered between subg. Hydnum and *Rufescentia*. In subg. Alba we recovered three monophyletic lineages that originate from eastern North America and correspond to phylogenetic species: *H.alboaurantiacum* sp. nov., *H.albidum*, and *H.albomagnum*. Subg. Pallida is represented by a single phylogenetic species from eastern North America: *H.subtilior* sp. nov. In subg. Hydnum three phylogenetic species from eastern North America were detected: *H.vagabundum* sp. nov., *H.washingtonianum*, and *H.subolympicum*. Subg. Rufescentia, centered around *H.rufescens*, contains the largest number of eastern North American phylogenetic species, including *H.ferruginescens* sp. nov., *H.aerostatisporum*, *H.canadense*, *H.mulsicolor*, *Hydnum* sp. AS30, *H.subconnatum* sp. nov., *H.quebecense*, *H.cuspidatum* sp. nov., and *H.umbilicatum*.

We were unable to produce ITS sequences from the holotypes of the following species: *H.albidum*, *H.albomagnum*, *H.umbilicatum* and *H.caespitosum*. As a result, we designated epitypes for three of those species – *H.umbilicatum* 10651TJB (CORT 012241), *H.albidum* 10640TJB (CORT 012029), and *H.albomagnum* RAS231 (TENN 073062), which are represented by ITS data. Each designated epitype was chosen based on morphological consistency and geographic proximity to sites containing holotypes. We were able to sequence partial ITS regions from two historical Peck collections labeled *H.albidum*, but based on morphology those specimens represent a separate, larger, white species that we describe here as *H.vagabundum*.

A total of sixteen species-level monophyletic groups from eastern North America were recovered, representing ten described and six undescribed species. One additional species, *H.geminum*, was not represented by any modern collections, and thus its phylogenetic position is unconfirmed. Below, taxonomic descriptions are presented for eastern North American species by subgenus. Of the recovered species, only one eastern North American *Hydnum*, *H.mulsicolor*, also occurs outside North America. This result is consistent with recent work that showed eastern North American clades of *Hydnum* are largely endemic (Feng et al. 2016, Niskanen et al. 2018).

None of the species studied display a clear preference for tree host genus. Many occupy a wide geographic range within eastern North America, with three species (*H.albidum*, *H.subtilior*, *H.vagabundum*) also found in Central America. One species, *H.washingtonianum*, occurs in both eastern and western North America.

Basidia in *Hydnum* were consistently suburniform, often undulating, and tapered to a narrow pedicel. Basidium shape was not considered a diagnostic trait and thus omitted from taxonomic descriptions below. Basidiospores of *Hydnum* were inamyloid and acyanophilous (Hall and Stuntz 1971). Morphological variation across *Hydnum* was low compared to other genera, with few variable microscopic features. As a consequence, species varied by differences in basidiospore size and shape, number of sterigmata, and pileipellis elements. Several taxa in subg. Rufescentia were nearly morphologically indistinct from one another and could be identified in the field only by a combination of morphology and distribution/habitat data. Even so, ITS sequencing is necessary to confidently identify those species.

### Taxonomy

#### Hydnumsubg.Alba Niskanen & Liimat., Mycologia 110: in press (2018)

##### Hydnum albidum

Taxon classificationFungiCantharellalesHydnaceae

Peck, Bulletin of the New York State Museum 1(2): 10 (1887)

Figs 3A, B , 6A

= Hydnumrepandumvar.albidum (Peck) Bres., Iconographia Mycologica 21: 1045 (1932)
= Dentinumalbidum (Peck) Snell, Mycologia 37: 51 (1945)
Hydnum = Hydnumrepandumf.albidum (Peck) Nikol., Flora Plantarum Cryptogamarum URSS. Fungi. Familia Hydnaceae 6(2): 306 (1961)

###### Type.

UNITED STATES. New York: Rensselaer County, Sandlake, ground in thin woods, Jul *ca.* 1886, C.H. Peck (holotype: NYS-F-134). **Epitype.** UNITED STATES. New York: Cortland County, Kennedy State Forest, Scutt Road (42.4685; -76.1656), on humus in forest with *Quercusrubra*, *Fagus*, *Acer*, 550 m, 30 Jul 2014, T.J. Baroni 10640TJB (CORT 012029, epitype here designated).

###### Description.

Pileus 15–50 mm wide, round to reniform, convex to plano-convex or uplifted, disc sometimes shallowly depressed; surface glabrous, sometimes irregularly bumpy or mottled in appearance, bright white becoming cream or cream-peach (10YR 8/4), no reaction to KOH; margin entire and incurved when young, undulating in age. Spines 1–6 mm long, easily rubbing off, subdecurrent to decurrent, white to cream white (10YR 8/3). Stipe 15–45 × 5–15 mm, central or eccentric, equal to slightly enlarged or bulbous at the base, then tapering into ground, concolorous with the pileus, staining orange-ochre (5A4–5B7 or “Yellow Ochre”). Basal mycelium white when present. Context white to pale cream, staining slowly orange (5A6) after five mins. Odor mild at first, then pleasantly fruity like apricots when stored in foil. Taste mild, pleasant, or occasionally peppery.

Basidiospores 4.5–*5.2*–6 μm × 3–*4*–4.5(5) μm, *Q*=(1.05)1.07–*1.33*–1.58(1.74) (n=72/5), subglobose to broadly ellipsoid, smooth, thin-walled, hyaline in KOH. Basidia 28–36(40) × 6–7(8) μm with 5–6(7) sterigmata. Pileipellis an interwoven cutis, hyphae smooth, cylindrical, thin-walled, mostly 3–6 μm wide. Clamp connections present.

###### Distribution.

Eastern Canada and U.S. and central Mexico – Nova Scotia, Vermont, New York (type), Tennessee, North Carolina. Also Veracruz, Mexico (GenBank KC152123).

###### Ecology.

In hardwood and mixed woods with *Betula*, *Quercus*, *Fagus*, *Tsuga*, *Pinus*, *Abies*. June to early September.

###### Other specimens examined.

CANADA. Nova Scotia: Victoria County, Cape North, Grey Glen Brook, Farm lot, with *Abies*, *Betula*, 80 m, 8 Sep 1973, R.H. Petersen TFB38198 (TENN 038198). UNITED STATES. New York: Cortland Co., Kennedy Forest, Scutt Hill Road, on humus under *Quercus*, *Fagus*, *Acer*, 550 m, 10 Aug 2003, T.J. Baroni 9623TJB (CORT 014489). North Carolina: Great Smoky Mountains National Park, Heintooga Round Bottom Road, top of road on embankment with *Betula*, *Picea*, 1525 m, 17 Aug 2017, R.A. Swenie RAS204 (TENN 071752). Great Smoky Mountains National Park, Heintooga Round Bottom Rd., on embankment with *Betula*, *Quercus*, *Tsuga*, 1525 m, 17 Aug 2017, R.A. Swenie RAS210 (TENN 073173). Tennessee: Great Smoky Mountains National Park, Ogle Place Nature Trail, near stream in soil among leaf litter with *Tsuga*, *Betula*, 670 m, 5 Jun 2016, R.A. Swenie RAS058 (TENN 072000). Great Smoky Mountains National Park, Schoolhouse Gap Trail, on embankment with *Quercus*, *Pinus*, *Betula*, 550 m, 8 Jul 2017, R.A. Swenie RAS158 (TENN 073041). Vermont: Windham County, Stratton Mountain Resort area, 650 m, 28 Jul 2017, T.J. Baroni 10788TJB (CORT 014475).

###### Discussion.

**Figure 3.**
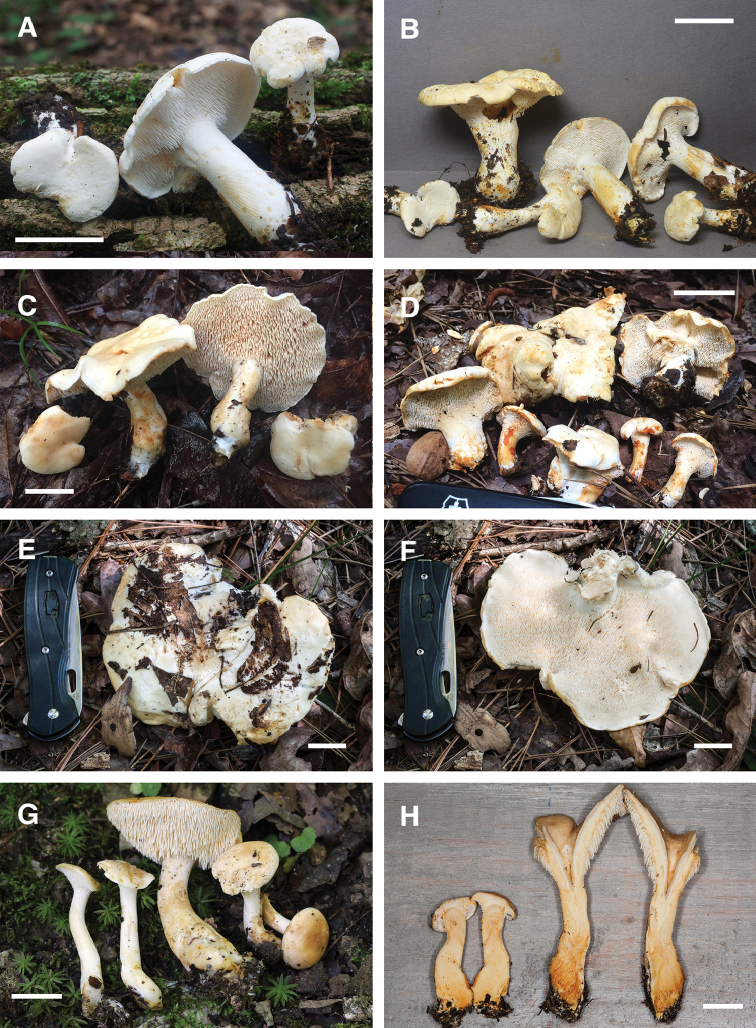
Basidiomes of *Hydnum* species. **A, B***H.albidum* 10640TJB (CORT 012029, epitype, photo T.J. Baroni) **C***H.alboaurantiacum* RAS186 (TENN 073053, holotype, photo R.A. Swenie) **D***H.alboaurantiacum* BPL876 (TENN 073003, photo B.P. Looney) **E, F***H.albomagnum* RAS231 (TENN 073062, epitype, photo R.A. Swenie) **G***H.subtilior* PBM4093 (TENN 073034, holotype, photo P.B. Matheny) **H***H.subtilior* showing characteristic staining of context RAS180 (TENN 073050, photo R.A. Swenie). Scale bar: 20 mm.

*Hydnumalbidum* was originally described from New York by Peck (1887) and produces small white to cream-colored basidiomes with small subglobose to broadly elliptic basidiospores. In the protologue Peck distinguishes *H.albidum* from *H.repandum* by the smaller basidiomes and spores, as well as white coloration. In a later description Peck (1897) added that *H.albidum* is an edible but uncommon species “uniformly colored in all its parts”. Of the two small white species of *Hydnum* that occur in eastern North America (Fig. 3A-D), both *H.albidum* and *H.alboaurantiacum* have similarly small subglobose basdiospores (Fig. 6A–B). However, *H.alboaurantiacum* quickly stains bright orange within minutes wherever handled, while *H.albidum* stains much less vividly brown-orange, sometimes only hours after handling. In addition, *H.alboaurantiacum* is only known from the southeastern US. While we were unable to successfully sequence DNA from the holotype of *H.albidum*, several collections from the region of the type locality are consistent with the morphology of the holotype of *H.albidum*, and one of these is designated as an epitype.

In addition to the holotype, there are several other historical collections made by Peck to which he applied the name *H.albidum*. Based on basidiospore measurements alone, it is clear three of the eight collections have much larger spores than *H.albidum*. We successfully sequenced partial ITS from two of those three collections, which matched modern specimens from Texas, New York, and Honduras belonging to a species more closely related to *H.repandum* (see discussion of *H.vagabundum*).

Hydnumrepandumvar.album (Quél.) Rea is a European variety, the name of which has been widely applied in North America (Coker and Beers 1951, Smith et al. 1981, Harrison and Grund 1987, Roody 2003). The description of H.repandumvar.album by Roody (2003) appears to refer to *H.subtilior*, while displaying a photo of what is perhaps *H.albidum*. However, the spores of *H.albidum* are smaller than the 7–8.5 × 5.5–7 μm listed by Roody (2003), Harrison and Grund (1987), Smith et al. (1981), and Coker and Beers (1951). Thus, the American concept of H.repandumvar.album is best interpreted as *H.subtilior*, described below.

##### Hydnum alboaurantiacum

Taxon classificationFungiCantharellalesHydnaceae

Swenie & Matheny sp. nov.

Figs 3C, D , 6B

###### Diagnosis.

Most similar to *Hydnumalbidum* but differs from it by the slightly stouter basidiomes that stain bright orange within minutes of handling. Differs from *H.subtilior* and *H.vesterholtii* by smaller basidiospores.

###### Type.

UNITED STATES. North Carolina: Great Smoky Mountains National Park, Smokemont, Bradley Fork Trail (35.5634; -83.3092), scattered under *Betula*, *Fagus*, with *Tsuga* nearby, 28 Jul 2017, R.A. Swenie RAS186 (holotype: TENN 073053).

###### Etymology.

*alboaurantiacum* (L.) white-orange, referring to the coloration of the basidiomes, which stain bright orange.

###### Description.

Pileus 20–70 mm wide, irregularly round, convex, becoming shallowly convex to depressed, occasionally umbilicate; margin thin, wavy to lobed, incurved becoming decurved; surface matt, glabrous, pale to cream white (“Pale Ochraceous Buff”), quickly bruising orange (“Zinc Orange” or “Xanthine Orange”, 6A6-8). Spines 1–7 mm long, brittle in mass and breaking easily, adnate to subdecurrent, white to cream-orange (“Pale Ochraceous Buff” to “Light Ochraceous Buff”, 4A2–5A3). Stipe 17–50 × 6–21 mm, central or eccentric, terete or clavate, concolorous with the pileus, easily bruising orange (5A2). Context thin, firm, cream white, staining orange (“Xanthine Orange” to “Mars Yellow”, 6A8 to 5B6–B7), especially in young specimens at base of stipe within five minutes when cut in half. Odor mild or sweet and fruity. Taste mild.

Basidiospores 4–*4.8*–6(7) μm × 3–*3.9*–5(6) μm, *Q*=1.00–*1.25*–1.52(1.54) (n=44/6), globose to ellipsoid, smooth, hyaline in KOH. Basidia 36–42 × 4.5–7 μm with 5–7 sterigmata. Pileipellis an interwoven cutis, hyphae smooth, cylindrical, thin-walled, mostly 3–5 μm wide. Clamp connections present.

###### Distribution.

Southeastern U.S. – North Carolina (type), Tennessee, and West Virginia (GenBank KU612600).

###### Ecology.

In mixed woods with *Quercus*, *Tsuga*, *Pinus*, *Betula*, *Liriodendron*, *Fagus*. May to August.

###### Other specimens examined.

UNITED STATES. North Carolina: Great Smoky Mountains National Park, Deep Creek, Indian Creek, on soil in mixed woods, 610 m, 9 Jul 1974, J.H. Restivo JHR1459 (TENN 040599). Great Smoky Mountains National Park, Smokemont, Bradley Fork Trail, scattered under *Betula*, *Fagus*, *Tsuga*, 700 m, 28 Jul 2017, R.A. Swenie RAS186 (TENN 073053). Great Smoky Mountains National Park, Heintooga Round Bottom Road, scattered on embankment with *Quercus*, *Betula*, *Tsuga*, 1525 m, 17 Aug 2017, R.A. Swenie RAS208 (TENN 071751). Macon County, vicinity of Highlands, Glen Falls Trail, 1100 m, 14 Jul 2000, E.B. Lickey TFB9833 (TENN 058812). Transylvania County, Pisgah National Forest, Yellow Gap Road, 310 m, 18 Jul 2000, R.H. Petersen TFB9764 (TENN 058665). Duke Forest, scattered in mixed duff with *Quercus*, *Pinus*, 150 m, 25 May 2016, B.P. Looney BPL876 (TENN 073003). Blue Ridge Parkway, near mile marker 342, side of road on embankment, deciduous woodlot, 1225 m, 19 Aug 2016, J. Schieb RAS104 (TENN 073014). Buncombe County, Bent Creek Experimental Forest, near Boyd Branch Road, mossy acidic forest with *Quercusalba*, *Q.rubra*, *Liriodendrontulipifera*, *Betulalenta*, *Tsugacanadensis*, 670 m, 22 Aug 2016, M. Hopping MH16004 (TENN 073548). Tennessee: Great Smoky Mountains National Park, Cades Cove, Gregory Ridge Trail, 550 m, 18 Aug 2005, E.B. Lickey TFB12761 (TENN 061328). Great Smoky Mountains National Park, Maddron Bald Trail, on mossy soil with *Tsuga*, *Quercus*, *Fagus*, *Pinus*, 550 m, 4 Aug 2012, S.A. Trudell SAT1221712 (TENN 067355). Sevier County, University of Tennessee Biology Field Station, on mossy soil with *Quercus*, *Tsuga*, *Pinus*, 450 m, 27 Jul 2009, A.D. Wolfenbarger AW0119 (TENN 064272).

###### Discussion.

*Hydnumalboaurantiacum* has probably been mistaken for the closely related *H.albidum* due to the initial pale white coloration and similar basidiospore size and shape. However, *H.alboaurantiacum* quickly stains bright rusty orange on all parts of the basidiomes where handled, whereas *H.albidum* slowly stains a lighter brown-orange hue. In addition, *H.alboaurantiacum* often displays a larger and more stout stature than *H.albidum*. The two species are readily distinguished as separate clades in Fig. 2. *Hydnumalboaurantiacum* is known only from the southeastern US and appears derived from a grade of Central American taxa including the recently described *H.zongolicense* (Niskanen et al. 2018).

##### Hydnum albomagnum

Taxon classificationFungiCantharellalesHydnaceae

Banker, Bulletin of the Torrey Botanical Club 28(4): 207 (1901)

Figs 3E, F , 6C

= Dentinumalbomagnum (Banker) Pouzar, Ceská Mykologie 10 (2): 76 (1956)

###### Type.

UNITED STATES. Alabama: Lee County, Auburn, Dec 1896, F.S. Earle (holotype: NY 776138). **Epitype.** UNITED STATES. Tennessee: Big South Fork National River & Recreation Area, Bandy Creek area (36.4920; -84.6950), solitary on soil with *Quercus*, *Pinus*, 450 m, 23 Sep 2017, RAS231 (TENN 073062, epitype here designated).

###### Description.

Pileus 60–110 mm wide, irregularly round, irregularly convex to plano-convex or uplifted; surface dull, glabrous with adhering debris, uneven and sometimes pitted in places, cream white (4A4) with patches of cottony white, becoming light tan in age (10 YR 6/4); margin thin, wavy to slightly lobed, incurved when young then raised in age. Spines 1–6 mm long, brittle in mass, adnate to subdecurrent, white to cream white (4A3–5A3). Stipe 20–40 × 13–20 mm thick, central or eccentric, clavate, occasionally split in two towards the apex; surface smooth, concolorous with spines, if bruising then only very slightly an hour or more after handling (“Yellow-Ocher” to “Ochraceous-Tawny”). Context fleshy, white, unchanging when cut. Odor mild or slightly acidic at first, then pleasantly fruity like apricots when stored in foil. Taste mild.

Basidiospores 5.5–*6.2*–7 μm × 3–*3.8*–5 μm, *Q*=1.24–*1.66*–2.07(2.17) (n=45/3), ellipsoid to broadly ellipsoid, smooth, thin-walled, hyaline in KOH. Basidia 38–46 × 5–6 μm with 4–5(6) sterigmata. Pileipellis a tightly interwoven cutis, hyphae smooth, cylindrical, thin-walled, mostly 2.5–5 μm wide. Clamp connections present.

###### Distribution.

Eastern U.S. – Massachusetts, North Carolina, Tennessee (epitype), and Alabama (holotype).

###### Ecology.

In hardwood and mixed woods with *Quercus*, *Pinus*. September to December.

###### Other specimens examined.

UNITED STATES. Massachusetts: Worcester County, Rutland State Park, gregarious under litter layer along edge of road with *Quercus*, *Pinusstrobus*, 260 m, 1 Nov 2003, P.B. Matheny PBM2512 (TENN 066858). North Carolina: Buncombe County, Black Mountain YMCA Blue Ridge Assembly, Wolfpit Loop, in leaf litter in broadleaf woods with *Quercus*, *Acer*, 900 m, 24 Sep 2015, B. Moerk NAMA 2015-050 (F C0305359F). Tennessee: Knox County, New Hopewell, on soil in mixed woods, 300 m, 2 Dec 1951, L.R. Hesler (TENN 020243).

###### Discussion.

The original description of *Hydnumalbomagnum* by Banker (1901) was based solely on dried material. As mentioned in the protologue, this species has a shorter stipe and overall stouter appearance than other *Hydnum*. In addition, mature basidiomes are much larger than other whitish small-spored species. *Hydnumalbomagnum* also has more strongly ellipsoid spores (*Q* values averaging 1.66) than other species. Historically, *H.albomagnum* has been less frequently collected than other pale species of *Hydnum*, perhaps because the basidiomes are often buried underneath layers of needle and leaf litter and thus overlooked. As basidiomes emerge from the ground, leaf litter remains stuck to the matt glabrous surface of the pileus, masking its appearance. This characteristic, along with the creamy white coloration, large basidiome size, and small ellipsoid spores, makes this one of the easier *Hydnum* species to identify. DNA sequencing of the type specimen of *H.albomagnum* was not successful; however, all modern collections reported under this species name have identical ITS sequences and match the morphology of the original species description. An epitype is chosen from Tennessee material, closest to the location of the holotype from Alabama.

Collections that are likely *H.alboaurantiacum* (TENN 041525) and *H.vagabundum* (TENN 003140) have been misidentified as *H.albomagnum*, perhaps due to the larger white appearance of those specimens. However, those collections differ from *H.albomagnum* in spore dimensions and lack litter debris on the pileus, which persists even after drying in *H.albomagnum*.

#### Hydnumsubg.Pallida Niskanen & Liimat., Mycologia 110: in press (2018)

##### Hydnum subtilior

Taxon classificationFungiCantharellalesHydnaceae

Swenie & Matheny sp. nov.

Figs 3G, H , 6D

Hydnum = Hydnumrepandumvar.album (Quél.) Rea sensu Am. auct.

###### Diagnosis.

*Hydnumsubtilior* is most closely related to European *H.vesterholtii* but differs from it based on ITS molecular data and geographic distribution in eastern North America.

###### Type.

UNITED STATES. Tennessee: Anderson County, Norris Dam State Park, Clear Creek Trail (36.2124; -84.0681), scattered on soil along trail under *Fagus*, *Carya*, *Quercus*, 24 Jun 2017, P.B. Matheny PBM4093 (holotype: TENN 073034).

###### Etymology.

*subtilior* (L.) finer, more slender, in reference to the slim basidiomes.

###### Description.

Pileus 20–90 mm wide, round or occasionally reniform, convex becoming plano-convex to depressed, sometimes umbilicate; surface matt, glabrous, sometimes cracking into scales at the center, light cream yellow to cream orange buff (“Marguerite Yellow” to “Light Ochraceous Buff”, 4A3–A5 to 5A2–A4), yellow with KOH, negative with FeSO4; margin thin, entire, incurved when young then decurved and sometimes wavy in age, staining rusty orange-brown (“Ochraceous-Orange” to “Mars Yellow”, 6A5 to 5B6–B7). Spines 1–8 mm long, adnexed to decurrent, cream white to pale orange-cream (5A1–A2). Stipe 20–60 × 5–21 mm, central or eccentric, sometimes curving, equal or enlarging towards base, cream white or slightly lighter than pileus, staining rusty orange-brown (5B6-B8). Context spongy, cream white to pale orange-cream, slowly staining orange (5A4–6) throughout after five minutes where cut. Odor mild or sweet. Taste mild or pleasant.

Basidiospores 7–*8*–9 μm × 5–*6.3*–7.5 μm, *Q*=1.07–*1.27*–1.52 (n=51/5), subglobose to broadly ellipsoid, smooth, thin-walled, hyaline in KOH. Basidia 32–44 × 7–9 μm with 3–5(6) sterigmata. Pileipellis an interwoven cutis. Hyphae smooth, cylindrical, thin-walled, mostly 3–7 μm wide. Clamp connections present.

###### Distribution.

Eastern U.S. – Illinois, North Carolina, Tennessee (type), Georgia, and Florida. Also Michoacán, Mexico (GenBank KY574324).

###### Ecology.

In hardwoods under *Quercus*, *Carya*, *Fagus*, *Carpinus* or in mixed woods with these trees or *Betula* and conifers such as *Tsuga* or *Pinus* or less frequently *Picea*. June to August.

###### Other specimens examined.

UNITED STATES. Florida: Alachua County, San Felasco Hammock Preserve State Park, Moonshine Sink Trail, in soil with deep layer of leaf litter, forest almost entirely *Carya*, 75 m, 23 Jul 2017, R.A. Swenie RAS180 (TENN 073050). Alachua County, Sweetwater Preserve off 16^th^ Street entrance, mixed hardwood forest of *Quercus*, *Carya*, *Carpinus* and occasionally *Pinus*, 35 m, 6 Aug 2017, B. Kaminsky & G. LaPierre (FLAS 61253). Georgia: Putnam County, Rock Eagle 4-H Camp, with *Quercus*, *Pinus*, *Carpinus*, 200 m, 20 Jul 2017, R.A. Swenie RAS170 (TENN 073049). Illinois: Coles County, Lakeview Park, scattered under *Quercusalba*, *Carya*, 215 m, 8 Aug 2009, M. Kuo MK08080904. North Carolina: Great Smoky Mountains National Park, Big Creek, Baxter Creek Trail to Mt. Sterling, on soil under *Tsuga*, 500 m, 9 Aug 2012, P.B. Matheny PBM3868 (TENN 067482). Great Smoky Mountains National Park, Smokemont, Bradley Fork Trail, scattered under *Betula*, *Fagus*, *Quercus*, *Tsuga*, 700 m, 28 Jul 2017, R.A. Swenie RAS184 (TENN 073051). Great Smoky Mountains National Park, Heintooga Round Bottom Road, solitary with *Betula*, *Picea*, 1525 m, 17 Aug 2017, R.A. Swenie RAS207 (TENN 073057). Tennessee: Great Smoky Mountains National Park, Tremont, Middle Prong Trail, scattered singly in mixed woods next to river with *Tsuga*, *Betula*, 450 m, 14 Jul 2013, P.B. Matheny PBM3923 (TENN 071999). Great Smoky Mountains National Park, Cades Cove Road, 610 m, 31 Jul 2004, R.H. Petersen TFB12107 (TENN 060045). Great Smoky Mountains National Park, Elkmont, solitary under *Quercus*, *Tsuga*, 670 m, 4 Aug 2009, J.M. Birkebak JMB080409-09 (TENN 064273). Great Smoky Mountains National Park, Tremont Institute, Lagoon Trail, solitary under *Tsuga*, *Carpinus*, *Betula*, 450 m, 23 Jun 2017, R.A. Swenie RAS148 (TENN 073035). Great Smoky Mountains National Park, Greenbrier picnic area, solitary in riparian forest under *Tsuga*, *Betula*, 500 m, 28 Jul 2017, B.P. Looney BPL987 (TENN 073032). Anderson County, Norris Dam State Park, Clear Creek Trail, solitary in litter on slope under *Quercus*, *Carya*, *Fagus*, 275 m, 31 Aug 2009, A.J. Floden AJF2 (TENN 069607).

###### Discussion.

*Hydnumsubtilior* is a common species in the southeastern U.S. found in deciduous and mixed forests with a variety of tree associates, often in deep layers of leaf litter. Environmental sequencing has recovered this species from *Quercus* root tips in central Mexico (García-Guzmán et al. 2017). The stipe is usually longer than the diameter of the pileus, and the overall coloration can range from light cream-yellow to peach or tan. The best diagnostic features for this species are the coloration and often elongated stature in combination with broadly ellipsoid spores averaging 8 × 6.3 μm. In addition, the context of fresh basidiomes stains orange throughout within five minutes when cut in half (Fig. 3H).

Earlier authors (Coker and Beers 1951, Smith et al. 1981, Harrison and Grund 1987, Roody 2003) referred to this species as H.repandumvar.album, a taxon originally described from Europe.

#### Hydnumsubg.Hydnum L.

##### Hydnum subolympicum

Taxon classificationFungiCantharellalesHydnaceae

Niskanen & Liimat., Mycologia 110: in press (2018)

Figs 4A, B , 6E

= Hydnumrepandum sensu Coker & Beers, 1951

###### Type.

CANADA. Newfoundland and Labrador: Near Humber Village, trail to Barry’s Lookout (48.9860; -57.7600), mature secondary growth of *Betulapapyrifera* and *B.alleghaniensis*, also with *Cantharellusamethysteus*, 2 Sept 2012, A. Voitk 12.09.02.av12 (holotype DAOM744368, isotype K(M)249002).

###### Description.

Pileus 80–130 mm wide, round, convex, becoming plano-convex; surface dry, glabrous, dull reddish-orange when young (5 YR 5/6) then cream to peach or dull orange in age (5A2-3), sometimes cracking in age to reveal white color of flesh; margin incurved and entire, becoming wavy and decurved, staining ochre to rusty brown very slowly after handling (“Yellow Ochre” to 5B8). Spines 1–7 mm long, close, subdecurrent, cream-yellow to pinkish cream. Stipe 30–100 × 20–40 mm, central or eccentric, tapering downwards to a slightly bulbous base, texture firm, smooth, white or off-white, staining orange cream to rusty orange, then yellow-brown when handled (“Mars Yellow”, 10YR 5/8). Context white to cream, dry, firm, brittle, discoloration not observed. Odor mild or fruity and reminiscent of apricots. Taste mild or slowly bitter or peppery.

Basidiospores 6–*7.5*–9 μm × 5–*6.1*–7 μm, *Q*=1.07–*1.23*–1.46 (n=38/3), subglobose to broadly ellipsoid, smooth, hyaline in KOH. Basidia 36–42 × 6.5–8 μm with (3)4–5 sterigmata. Pileipellis an interwoven cutis, hyphae smooth, cylindrical, thin-walled, mostly 4–6 μm wide. Clamp connections present.

###### Distribution.

Eastern North America – Newfoundland and Labrador (type), Quebec (GenBank No. KM406979), Maryland, Virginia, North Carolina, Tennessee, and Georgia.

###### Ecology.

In hardwood or mixed woods with *Quercus*, *Betula*. August to October.

###### Other specimens examined.

UNITED STATES. Georgia: White County, Unicoi State Park, Unicoi to Helen Trail, hidden in soil embankment under roots, 460 m, 16 Jul 2017, R. Healy RAS168 (TENN 073047). Maryland: Harford County, Susquehanna State Park, 60 m, 9 Sep 2016, RAS118 (TENN 073021). North Carolina: Buncombe County, Bent Creek Experimental Forest, Boyd Branch, solitary in mature bottomland forest including *Liriodendrontulipifera*, 670 m, 19 Sep 2016, M. Hopping MH16009 (TENN 073551). Tennessee: Great Smoky Mountains National Park, Schoolhouse Gap Trail, solitary in mixed woods under *Quercus*, 490 m, 26 Oct 2013, A.J. Ramsey AJR14 (TENN 073004). Virginia: Grayson County, Mount Rogers National Recreation Area, Mt. Rogers Trail, scattered along trail in mixed hardwood forest with *Betula*, *Quercus*, 1225 m, 17 Aug 2015, R.A. Swenie RAS029 (TENN 070845).

###### Discussion.

This eastern North American species is phylogenetically allied with *H.repandum* and relatives in subgenus Hydnum along with *H.vagabundum* sp. nov. (described below) and *H.washingtonianum*. It can be distinguished from European *H.repandum* mainly by the different geographic distribution (eastern North America). This species differs from *H.vagabundum* by the smooth yellow-peach pileus that tends to crack in age and mostly non-lobate pileus margin. In comparison to *H.washingtonianum*, *H.subolympicum* produces smaller spores. Because of the shape and coloration, basidiomes of *H.subolympicum* in the field can resemble large chanterelles from above. Like many other species of *Hydnum*, basidiomes of this species often possess the sweet apricot-like odor that is characteristic of chanterelles. In our experience, this species is a choice edible. Coker and Beers (1951) likely referred to this taxon under the name *H.repandum*.

**Figure 4.**
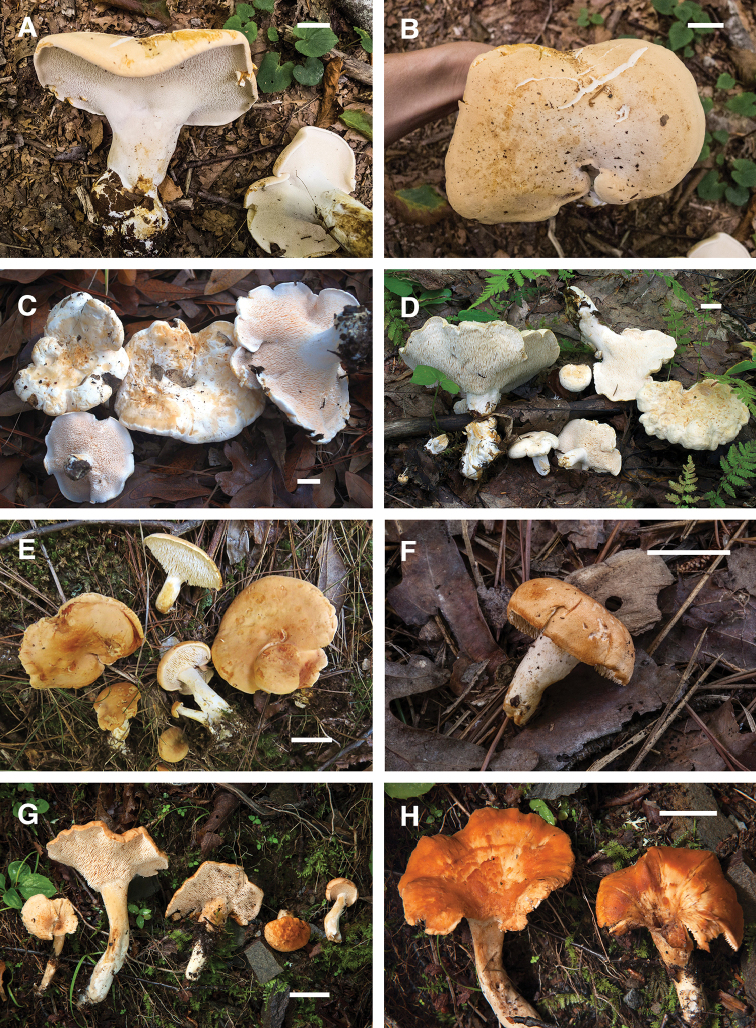
Basidiomes of *Hydnum* species. **A, B***H.subolympicum* RAS029 (TENN 070845, photo R.A. Swenie) **C***H.vagabundum* CLO4985 (CSU 01477, holotype, photo C.L. Ovrebo) **D***H.vagabundum* 10782TJB (CORT 014461, photo T. J. Baroni) **E***H.ferruginescens* MH16005 (TENN 073549, holotype, photo M. Hopping) **F***H.ferruginescens* RAS229 (TENN 073061, photo R.A. Swenie) **G, H***H.aerostatisporum* RAS071 (TENN 073001, photo R.A. Swenie). Scale bar: 20 mm.

##### Hydnum vagabundum

Taxon classificationFungiCantharellalesHydnaceae

Swenie, Ovrebo & Matheny sp. nov.

Figs 4B, C , 6F

###### Diagnosis.

Closely related to *Hydnumsubolympicum* but differs from it by the paler, more lobate pileus and ITS sequence divergence.

###### Type.

UNITED STATES. Texas: Newton County, State Highway 87 and County Road 3062 (30.7080; -93.8270), scattered in soil under *Fagus*, *Pinus*, *Quercus*, 29 Dec 2011, C.L. Ovrebo CLO4985 (holotype: TENN 074443).

###### Etymology.

*vagabundum* (L.), wandering, roving about, in reference to the broad distribution of this species in North America.

###### Description.

Pileus 30–140 mm wide, round, convex, becoming plano-convex to broadly depressed; margin incurved and often lobed when young, then decurved or straight and wavy in age; surface matted tomentose or glabrous and pitted-grooved to bumpy in areas, off-white with pale pinkish buff tones (5A2), sometimes with slight ochre hues (5C4–C5), staining ochre where bruised. Spines 1–12 mm long, shortest near the pileus margin, adnate to subdecurrent, concolorous with the pileus or slightly darker pinkish-orange (5A3). Stipe 20–60 × 10–30 mm, central or eccentric, equal or with a swollen base, surface smooth or soft matted-tomentose, white or concolorous with the pileus, pinkish tan in areas, slowly staining ochre where bruised. Context solid, white, discoloring slight ochre (5C4–C5) where cut in half. Odor not distinctive. Taste mild or sweet-nutty, then slowly slightly acidulous.

Basidiospores 6.5–*7.4*–8.5 × 5–*6.1*–7.5 μm, *Q*=1.03–*1.22*–1.43(1.60) (n=97/4), subglobose to broadly ellipsoid, smooth, thin-walled, hyaline in KOH. Basidia 39–57 × 8–10.5 μm with (3)4–5 sterigmata. Pileipellis an interwoven cutis, hyphae smooth, cylindrical, thin-walled, mostly 3–7 μm wide. Clamp connections present.

###### Distribution.

Eastern U.S. and Central America – New York, Texas (type), Honduras (GenBank HM639267–HM639268).

###### Ecology.

In mixed woods with *Fagus*, *Pinus*, *Quercus*, *Tsuga*, *Picea*, *Betula*. May to December.

###### Other specimens examined.

UNITED STATES. New York: Bolton, Sand Lake, August, C.H. Peck (NYS-D-7819). Rensselaer County, Burden Lake, 1 Sep, C.H. Peck (NYS-D-7820). Tompkins County, Hammond Hill State Forest, Red Man Run Rd., on humus in mixed woods with *Tsuga*, *Fagus*, *Picea, Betulaalleghaniensis*, 520 m, 1 Sep 2017, T.J. Baroni 10782TJB (CORT 014461).

###### Discussion.

*Hydnumvagabundum* is a large whitish species in subgenus Hydnum. Two collections of this species at NYS from the late 1800s and early 1900s were misidentified as *H.albidum* by Peck but feature distinctly larger basidiospores than the holotype of *H.albidum*. In addition, basidiomes of this species are much larger and fleshier than those of *H.albidum*. Partial ITS sequences obtained from Peck’s specimens match two extant collections from New York and Texas, as well as GenBank sequences from western Honduras (HM639268, HM639267; Sarmiento and Fontecha 2013). In Honduras this species can be found in May and June, where it is a common edible (Sarmiento and Fontecha 2013). To date, *H.vagabundum* has the widest known range among the endemic eastern North American *Hydnum* species.

Peck’s notes of what is described here as *H.vagabundum* indicate it as a “white, edible *Hydnum*”. Another large whitish species, *H.albomagnum*, can be distinguished from *H.vagabundum* by the copious amount of leaf and needle debris that adheres to the pileus surface and smaller basidiospores.

##### Hydnum washingtonianum

Taxon classificationFungiCantharellalesHydnaceae

Ellis & Everhart, Proc. Phila. Acad. 1894: 323 (1894)

= Hydnumneorepandum Niskanen & Liimat., Mycologia 110: in press (2018)

###### Type.

UNITED STATES. Washington: Kitsap County, Tracyton (47.6090; -122.6540), on ground in deep coniferous woods, 27 Dec 1893, A.M. Parker (holotype: NY 776185, isotype: WTU-F-14341).

###### Description.

Pileus up to 40 mm wide, subplane, slightly depressed, thin, irregular; surface glabrous, “subviscose”, wrinkled when dry, pale orange. Spines 3–5 mm long, terete, slender, acute, decurrent half way down the stipe, pale yellow but nearly white when fresh. Stipe up to 40 × 5–10 mm, subcylindrical, tapering slightly towards the base, central or slightly eccentric, pale orange. Context fleshy.

Basidiospores 7–*7.7*–8.5 μm × 6–*6.8*–7.5(8) μm, Q=1.04–*1.13*–1.22 (n=40/2), subglobose to broadly ellipsoid, smooth, thin-walled, hyaline in KOH. Basidia 31–41 × 7.5–8.5 μm with 4 sterigmata. Pileipellis hyphae not reviving. Clamp connections present.

###### Distribution.

Western North America and eastern Canada – British Columbia, Washington (type), California (GenBank GU180269, MG972632), and Newfoundland and Labrador.

###### Ecology.

On ground in coniferous woods. December.

###### Discussion.

*Hydnumwashingtonianum*, originally described from the Puget Sound region of Washington, is characterized by the pale orange pileus, yellowish decurrent spines, small globose basidiospores, and tough flesh. The species was considered synonymous with *H.repandum* by Maas Geesteranus (1964) and Hall and Stuntz (1971). However, we were able to produce a partial ITS sequence from the isotype (GenBank MH379846), which does not match European *H.repandum* sequences. Thus, we consider this species as an autonomous taxon with a mostly northern geographic distribution in North America. Phylogenetic analysis of the ITS sequence confirms this species from Washington, British Columbia, California, and Newfoundland and Labrador. *Hydnumwashingtonianum* is associated with coniferous forests on both coasts, and one environmental sequence (GenBank GU180269) recovered this species on root tips of *Pinusmuricata* in California.

*Hydnumneorepandum*, a recently described species from Newfoundland and Labrador (Niskanen et al. 2018), has an ITS sequence that differs by a single base pair from that of the isotype of *Hydnumwashingtonianum*. The morphology of both protologues is also in agreement. Thus, we consider *H.neorepandum* a taxonomic synonym of *H.washingtonianum*.

#### Hydnumsubg.Rufescentia Niskanen & Liimat., Mycologia 110: in press (2018)

##### Hydnum ferruginescens

Taxon classificationFungiCantharellalesHydnaceae

Swenie & Matheny sp. nov.

Figs 3D , 5G

###### Diagnosis.

Most closely related to the Eurasian *Hydnummagnorufescens* but differs from it by somewhat smaller basidiospores, ITS sequence divergence, and geographic distribution in the southeastern U.S.

###### Type.

UNITED STATES. North Carolina: Buncombe County, Tanbark Ridge (35.6535; -82.4853), growing singly or conjoined in moss along trail with *Pinusstrobus*, *Quercusprinus*, *Kalmialatifolia*, 915 m, 4 Sep 2016, M. Hopping MH16005 (holotype: TENN 073549).

###### Etymology.

*ferruginescens* (L.), becoming ferruginous or rust-colored, in reference to the overall coloration of this species.

###### Description.

Pileus 22–60 mm wide, round, convex, becoming depressed; margin incurved and entire when young, then irregularly lobed or degraded in age; surface dry, glabrous, tawny to fulvous (5YR 5/8 to 6/8), discoloring slightly darker when handled. Spines 1–4 mm long, shorter near the margin, adnate to subdecurrent, white to cream (5A2–A4), bruising orange. Stipe 15–40 × 5–12 mm, central or eccentric, equal or slightly wider at apex, texture smooth, white or cream, lightly bruising orange (7.5 YR 7/8, 5A6-7); thick white mycelial mat sometimes present at base of stipe. Context white, unchanging after 5 minutes where cut in half. Odor not distinctive. Taste not distinctive or mildly fruity.

Basidiospores (5.5)6–*6.9*–8 μm × 5–*6.3*–7.5 μm, *Q*=1.01–*1.09*–1.22, (n=45/2), globose to subglobose, smooth, hyaline in KOH. Basidia 39–56 × 7.5–9 μm with (3)4–5 sterigmata. Pileipellis an interwoven cutis, hyphae smooth, cylindrical, thin-walled, mostly 5–7 μm wide. Clamp connections present.

###### Distribution.

Southeastern U.S. – North Carolina (type), Tennessee, Arkansas (GenBank KX358033).

###### Ecology.

In mixed woods with *Quercus*, *Pinus*, *Carya*, *Tsuga*. September.

###### Other specimens examined.

UNITED STATES. Tennessee: Big South Fork National River & Recreation Area, West Bandy Trail, scattered along trail with *Quercus*, *Carya*, *Pinus*, *Tsuga*, 450 m, 23 Sep 2017, R.A. Swenie RAS229 (TENN 073061).

###### Discussion.

*Hydnumferruginescens* is known only from three occurrences in the southeastern U.S. This species is similar to *H.magnorufescens*, which has similarly sized basidiomes but slightly larger spores and is known from Europe and Asia.

**Figure 5.**
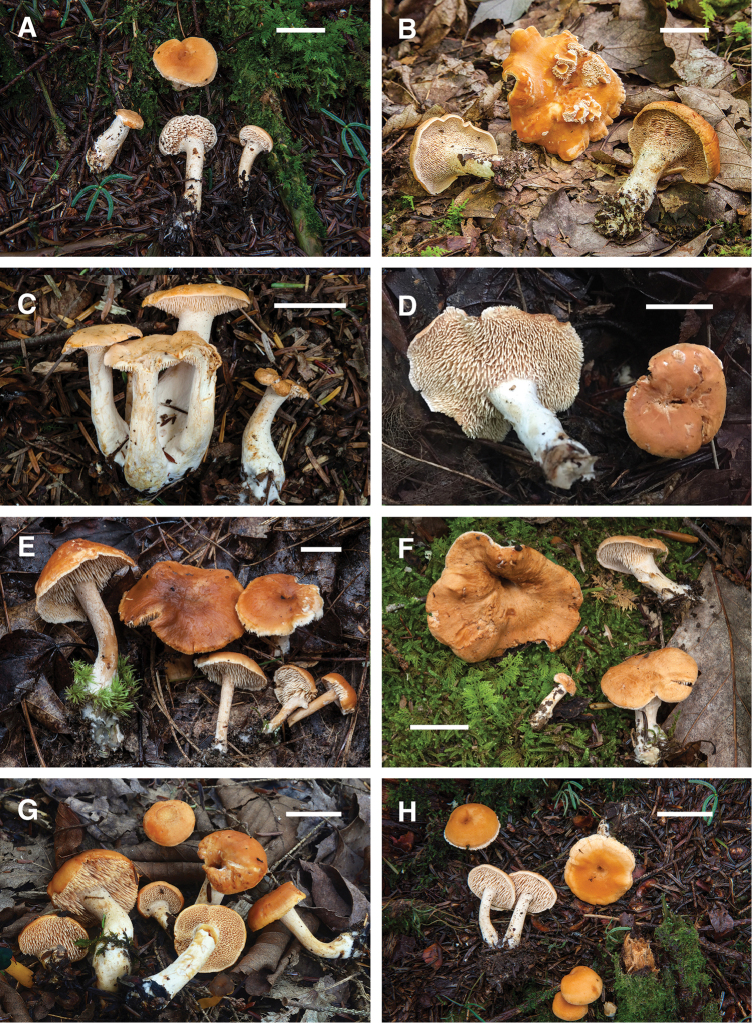
Basidiomes of *Hydnum* species. **A***H.canadense* RAS100 (TENN 073010, photo R.A. Swenie) **B***H.mulsicolor* RAS023 (TENN 070321, photo R.A. Swenie) **C***H.subconnatum* RAS235 (TENN 073064, holotype, photo R.A. Swenie) **D***H.subconnatum* RAS169 (TENN 073048, photo R.A. Swenie) **E***H.cuspidatum* RAS246 (TENN 073086, holotype, photo R.A. Swenie) **F***H.cuspidatum* RAS150 (TENN 073037, photo R.A. Swenie) **G***H.umbilicatum* 10651TJB (CORT 012241, epitype, photo T.J. Baroni) **H***H.umbilicatum* RAS101 (TENN 073011, photo R.A. Swenie). Scale bar: 20 mm.

##### Hydnum aerostatisporum

Taxon classificationFungiCantharellalesHydnaceae

Buyck, Lewis & V. Hofstetter, Crypt. Mycologie, 38: 101–146 (2017)

Figs 3E, F , 5H

= Hydnumsubrufescens Niskanen & Liimat., Mycologia 110: in press (2018)

###### Type.

UNITED STATES. Texas: Polk County, Big Thicket Natural Preserve, Big Sandy Creek Unit, Beaver Slide Trail (30.6150; -94.6700), 4 Jul 2014, Buyck 14.156 (PC0142475).

###### Description.

Pileus (20)30–100 mm wide, irregularly round or sometimes reniform, convex to plano-convex, becoming funnel-shaped in age, sometimes with slit or umbilicus forming over stipe, surface dry, glabrous, subzonate when young, then cracking to coarsely scurfy in age, bright to medium brownish orange (“Xanthine Orange” to “Orange Rufous”), paler when young (“Salmon-Orange”), often cracking in age to reveal lighter color of context (“Pale Pinkish Buff”); margin incurved and entire when young, then wavy, irregular or degraded in age, discoloring slightly darker after handling. Spines 1–9 mm long, close, mostly awl-shaped but occasionally spathulate, adnate to subdecurrent, buff to peach (“Light Buff” to “Pinkish Buff”). Stipe 25–80 × (3)7–25 mm, central or eccentric, equal or slight bulbous at base in younger specimens, smooth, often with white hazy or cottony patches overlaid on surface, cream white to pale orange in younger basidiomes, then darker tan orange with age, discoloring very slightly brownish orange when handled. Context cream to peach colored, firm, sometimes hollow with age, unchanging after 5 minutes when cut in half. Odor mild or sweet at first, then pleasantly fruity like apricots when stored in foil. Taste mild or weakly acrid.

Basidiospores 7–*8.1*–8.5 × 6–*7*–8 μm, *Q*=1.01–*1.15*–1.33 (n=38/3), mostly broadly ellipsoid, smooth, hyaline in KOH. Basidia 40–47 × 8–10 μm with (2)3–5 sterigmata. Pileipellis an interwoven cutis, hyphae smooth, cylindrical, thin-walled, mostly 4–6 μm wide. Clamp connections present.

###### Distribution.

Eastern U.S. – Illinois, Texas (type), North Carolina, Tennessee, Virginia, and Florida.

###### Ecology.

In hardwoods of *Quercus*, *Carya*, *Ulmus* or mixed woods including *Betula*, *Picea*, *Tsuga*. June to October.

###### Specimens examined.

UNITED STATES. Florida: Alachua County, Gainesville, University of Florida Natural Area Teaching Laboratory, 30 m, 9 Sept 2016, A. I. Zuniga AIZ-021 (FLAS 60406). Alachua County, Gainesville, Possum Creek Park, in deep woods with *Carya* and some *Quercus*, soil rich and not sandy, 55 m, 9 Oct 2015, M.E. Smith MES1432 (FLAS 59996). Illinois: Coles County, Fox Ridge State Park, 230 m, 28 Sep 1996, M. Kuo MK09289621. Dewitt County, Weldon Springs State Recreation Area, gregarious under *Carya* with *Quercusalba*, *Quercusrubra*, *Ulmus* nearby, 215 m, 18 Aug 2014, M. Kuo MK09181403. North Carolina: Great Smoky Mountains National Park, Heintooga Round Bottom Road, scattered on mossy bank with *Picea*, *Betula*, other hardwoods, 1580 m, 24 Jul 2016, R.A. Swenie RAS071 (TENN 073001). Great Smoky Mountains National Park, Heintooga Round Bottom Road, solitary on embankment with *Picea*, *Betula*, 1525 m, 17 Aug 2017, R.A. Swenie RAS211 (TENN 073174). Great Smoky Mountains National Park, Cataloochee, Big Fork Ridge Trail, 1100 m, 18 Jun 2005, E.B. Lickey TFB12514 (TENN 060681). McDowell County, near Little Switzerland, with *Tsuga*, ca. 1000 m, 19 Aug 2016, A. Funston RAS107 (TENN 073017). Tennessee: Great Smoky Mountains National Park, Cades Cove Road, 1 mile before Schoolhouse Gap Rd, 610 m, 31 Jul 2004, R.H. Petersen TFB12108 (TENN 060046). Great Smoky Mountains National Park, Tremont River Trail, solitary on slope in mossy area in mixed woods with *Tsuga* and hardwoods, 450 m, 4 Jul 2017, B. Teresi & K. Hucks RAS157 (TENN 073040). Texas: Polk County, Big Thicket National Preserve, Beaverslide Trail, on ground in mixed woods, 30 m, 12 Jun 2017, R.L. Pastorino RLP61217D (TENN 073547). Virginia: Shenandoah National Park, Hogback Mountain, 600 m, 9 Sept 2016, RAS121 (TENN 073024).

###### Discussion.

*Hydnumaerostatisporum* is a commonly encountered species in the eastern U.S. It has been found primarily in hardwoods and mixed woods including conifers at high and low elevations on both sandy and non-sandy soils. The vibrant medium to dark orange pileus transitions from smooth in young specimens to conspicuously cracked and scurfy in age, often becoming funnel-shaped, occasionally with a hole or umbilicus. The stipe frequently becomes darker tan-orange in age, which is unusual in other medium-sized orange-pileate species of *Hydnum*. Basidiomes, particularly older specimens, often have patches of hazy white on the stipe surface.

*Hydnumaerostatisporum* was recently re-described as a new species from Quebec – *H.subrufescens* (Niskanen et al. 2018). The ITS sequence of the holotype of *H.subrufescens* differs from that of the holotype of the earlier described *H.aerostatisporum* by seven base pairs, but *H.subrufescens* does not form a well-supported monophyletic group in our phylogenetic analyses and recognition of *H.subrufescens* as a separate species would render *H.aerostatisporum* paraphyletic (Fig. 2). The morphology of both is consistent, including the similarly sized globose to subglobose spores. For these reasons, we consider *H.subrufescens* a taxonomic synonym of *H.aerostatisporum*.

##### Hydnum canadense

Taxon classificationFungiCantharellalesHydnaceae

Niskanen & Liimat., Mycologia 110: in press (2018)

Figs 3G , 5I

###### Type.

CANADA. Newfoundland and Labrador: Near Grand Falls, south of the Exploits River, west of Hwy 360, south of Hwy 1, along a gravel road beside Moccasin Lake (48.9030; -55.5580), in conifer-dominated forest, 9 Sep 2009, K. Liimatainen & T. Niskanen 09-006 (holotype H7043727, isotype K(M)248978, isotype NY).

###### Description.

Pileus 12–25 mm wide, irregularly round to slightly reniform, convex to plano-convex, surface dry, glabrous, orange (“Zinc Orange” to “Xanthine Orange”), sometimes cracking in age near central depression; margin incurved and entire or slightly degraded. Spines 1–3 mm long, adnate, cream-colored, at times thick and somewhat flattened. Stipe 15–35 × 5–8 mm, central or eccentric, equal or widening at base, firm, smooth, white to cream, lightly staining ochre to medium brownish orange (“Mars Yellow” to “Orange Rufous”) where handled. Context not observed. Odor and taste mild.

Basidiospores 7–*8*–9(9.5) μm × 7–*7.6*–9 μm, *Q*=1.00–*1.05*–1.11, (n=38/1), globose to subglobose, smooth, hyaline in KOH. Basidia 38–46 × 7.5–9.5 μm with 3–5 sterigmata. Pileipellis an interwoven cutis, hyphae smooth, cylindrical, thin-walled, mostly 5–7 μm wide. Clamp connections present.

###### Distribution.

Eastern North America – Newfoundland and Labrador (type, GenBank KX388681) and North Carolina.

###### Ecology.

In conifer forest with *Abiesfraseri*. August to September.

###### Specimen examined.

UNITED STATES. North Carolina: Yancey County, Mount Mitchell State Park, in fir forest, 2000 m, 19 Aug 2016, R.A. Swenie RAS100 (TENN 073010).

###### Discussion.

*Hydnumcanadense* is only known from high-elevation and higher latitude conifer forests in North Carolina and eastern Canada. The North Carolina collection was found among several basidiomes of *H.umbilicatum*. *Hydnumcanadense* can be distinguished from *H.umbilicatum* by the 3–5 sterigmata (versus 2–4 sterigmata in *H.umbilicatum*) and ITS sequence divergence. *Hydnumcanadense* differs from the closely related *H.mulsicolor* by the association with conifers and larger spore size. Another closely related species, *H.submulsicolor*, is morphologically indistinguishable from *H.canadense* according to Niskanen et al. (2018), and ITS sequencing is likely necessary to reliably differentiate it from *H.canadense*. The spores of the North Carolina collection of *H.canadense* reported here are more globose with a lower average *Q* value than that of the Newfoundland and Labrador collections reported by Niskanen et al. (2018).

##### Hydnum mulsicolor

Taxon classificationFungiCantharellalesHydnaceae

Liimat. & Niskanen, Mycologia 110: in press (2018)

Figs 3H , 5J

###### Type.

SLOVENIA. ﻿Velike Lašče (45.8500; 14.6000): ﻿In forest of *Piceaabies*, *Fagussylvatica*, and *Corylusavellana*, GIS 1336 (holotype LJF1057).

###### Description.

Pileus 30–45 mm wide, round, convex when young, becoming plano-convex to funnel-shaped; surface dry, glabrous or matted-tomentose, bright orange to tan (“Zinc Orange” to “Ochraceous-Tawny”) becoming subzonate towards margin, sometimes distinctly umbilicate at center; margin incurved at first, becoming decurved, wavy, and lightening in color. Spines 1–7mm long, decurrent, pinkish cream with white tips. Stipe 25–45 × 5–8 mm, central or eccentric, equal or enlarging downwards, texture firm, smooth, with aborted spines at stipe apex and some texturing below, cream white, sometimes with small white cottony patches, staining orange when handled, a dense mat of basal mycelium present at base. Context not observed. Odor mild or pleasant. Taste not distinctive.

Basidiospores 6.5–*7.7*–8.5 μm × (5.5)6–*7.1*–8.5 μm, *Q*=1.00–*1.08*–1.19(1.24) (n=46/3), subglobose, smooth, hyaline in KOH. Basidia 52–60 × 7.5–9.5 μm with 3–4(5) sterigmata. Pileipellis an interwoven cutis, hyphae smooth, cylindrical, thin-walled, mostly 4–6 μm wide. Clamp connections present.

###### Distribution.

Eastern U.S. and Central Europe – Ohio, Virginia, North Carolina, Tennessee (GenBank AJ547885, AJ547868, AJ783969), and Slovenia (type), Switzerland (GenBank KU612545, KX086216).

###### Ecology.

In deciduous or mixed forests with *Quercus*. July to September.

###### Other specimens examined.

UNITED STATES. North Carolina: Blue Ridge Parkway near Little Switzerland, deciduous woodlot with *Quercus*, 1100 m, 19 Aug 2016, M. Hopping RAS105 (TENN 073015). Blue Ridge Parkway near Little Switzerland, in mixed woods, 1100 m, 19 Aug 2016, D. Boes RAS108 (TENN 073018). Buncombe County, Tanbark Ridge, growing singly in mature acidic cove forest with *Quercus* and *Acer*, 915 m, 4 Sep 2016, M. Hopping MH16006 (TENN 073550). Tennessee: Great Smoky Mountains National Park, Cherokee Orchard, Bullhead Trail, on soil in mixed forest, 1340 m, 16 Jul 2015, R.A. Swenie RAS023 (TENN 070321). Virginia: Shenandoah National Park, milepost 21, 1000 m, 9 Sep 2016, RAS120 (TENN 073023).

###### Discussion.

*Hydnummulsicolor* is the only species of *Hydnum* in eastern North America that is also known to occur in Europe based on ITS phylogenetic analysis. The basidiomes are small to medium-sized with strongly decurrent spines, and the pileus color ranges from strikingly orange to tan. Prominent basal mycelium is also present as a dense mat or as distinct rhizomorphs at the stipe base. In the eastern US, *H.mulsicolor* is often found with *Quercus* in mixed or hardwood forests. It is closely related to *H.submulsicolor* and *H.canadense*, both of which are known only from coniferous forests in eastern North America and have slightly larger spores than *H.mulsicolor* (Niskanen et al. 2018).

##### Hydnum quebecense

Taxon classificationFungiCantharellalesHydnaceae

Niskanen & Liimat., Mycologia 110: in press (2018)

Fig. 5K

###### Type.

CANADA. Québec: ﻿Saint-Donat (46.3000; -74.2000), in conifer-dominated forest (*Tsuga*, *Abies*, *Picea*, *Betula*, and *Populus*), 5 Sep 2010, anonymous, T. Niskanen 10-064 (holotype H7043948, isotype K(M)248983, isotype NY).

###### Description.

Pileus 2–20 mm wide, round or sometimes irregularly so, convex, apex sometimes depressed or umbilicate, surface dry, tomentose or velutinous, tan orange-brown to warm reddish-brown; margin incurved, becoming decurved and wavy. Spines 1–2 mm long, adnate when young, subdecurrent with age, cream-white to peach. Stipe 15–45 × 3–10 mm, central, equal to subclavate, glabrous to minutely velutinous, cream white to very light buff orange, staining buff orange when handled. Context solid, cream to tan. Odor and taste mild.

Basidiospores 8–*8.4*–9.5 μm × 7–*7.8*–9 μm, *Q*=1.00–*1.09*–1.28 (n=19/2), globose to subglobose, smooth, hyaline in KOH. Basidia 40–51 × 7–8 μm with 2–3 sterigmata. Pileipellis an interwoven cutis, hyphae smooth, cylindrical, thin-walled, hyaline, mostly 5–8 μm wide. Clamp connections present.

###### Distribution.

Eastern U.S. and eastern Canada – New York, Quebec (type, GenBank KX388662).

###### Ecology.

In moss, especially *Sphagnum*, under *Fagus* and *Picea*. July.

###### Other specimens examined.

UNITED STATES. New York: Hamilton County, Raquette Lake, Long Point, Blue Mountain Trail, in moss under *Fagusgrandifolia*, 550 m, 25 Jul 2001, J. D’Apice 49JD (CORT 7367). Hamilton County, Raquette Lake, Silver Beach Bog, in *Sphagnum*, 550 m, 22 Jul 1997, J. Guardino JG003 (CORT 7330). Hamilton County, Raquette Lake, Silver Beach Bog, in *Sphagnum*, 550 m, 23 Jul 1988, C. Nelson CN9 (CORT 7365). Hamilton County, Raquette Lake, Silver Beach Bog, in *Sphagnum* under *Picea*, 550 m, 26 July 1993, K. Hodge KH7 (CORT 7322).

###### Discussion.

This species is closely related to *H.umbilicatum* but is characterized by the apparent habitat preference for *Sphagnum* bogs. The holotype from Quebec (KX388662) was described among *Sphagnum* in association with conifers and northern hardwoods (mainly *Picea*, but also *Tsuga*, *Abies*, *Betula*, *Populus*).

##### Hydnum

Taxon classificationFungiCantharellalesHydnaceae

sp. AS30

###### Description.

Pileus 20 mm wide, round, umbilicate, deep buff. Stipe less than 10mm long, central.

Basidiospores 6.5–*7.2*–8 μm × 6–*6.9*–7.5 μm, *Q*=1.00–*1.05*–1.1 (n=12/1), globose to subglobose, smooth, hyaline in KOH. Basidia 44–48 × 8–10.5 μm with (2) 3–4 sterigmata. Pileipellis an interwoven cutis, hyphae smooth, cylindrical, thin-walled, hyaline, mostly 5–7 μm wide. Clamp connections present.

###### Distribution.

Eastern U.S. – New York.

###### Ecology.

In *Sphagnum*, under *Tsuga* and *Larix*. July.

###### Specimen examined.

UNITED STATES. New York: Hamilton County, Raquette Lake, Silver Beach Bog, *Sphagnum* substrate under *Tsuga*, *Larix*, 550 m, 28 Jul 1982, A. Sabol AS30 (CORT 007356).

###### Discussion.

This species is known only from a single basidiome collected in New York. The description is drawn from the original collection notes. It is closely related to *H.subconnatum* but differs from it by the smaller spore size, shorter stipe, and association with *Sphagnum*. It is recorded from the same locality and habitat as *H.quebecense* but differs from that species by ITS sequence and smaller spores. We refrain from describing the taxon as new until confirmed by additional collections and sequence data.

##### Hydnum subconnatum

Taxon classificationFungiCantharellalesHydnaceae

Swenie & Matheny sp. nov.

Figs 4A , 5L

###### Diagnosis.

Closely related to *Hydnumoregonense* but differs from it by ITS sequence divergence and geographic distribution. *Hydnumsubconnatum* is known only from the southeastern U.S.

###### Type.

UNITED STATES. North Carolina: Yancey County, Carolina Hemlocks Recreation Area, picnic area (35.8057; -82.2047), on soil growing in fused cluster and singly with *Tsugacarolinensis*, *Quercus*, *Liriodendron*, 840 m, 29 Sep 2017, R.A. Swenie RAS235 (holotype: TENN 073064).

###### Etymology.

*subconnatum* (L.), born together, in reference to the fused stipe bases of multiple basidiomes.

###### Description.

Pileus 10–75 mm wide, more or less round, broadly convex to plane, surface usually dry but sometimes slightly hygrophanous, glabrous, occasionally with shallow cracks or pits in age, sometimes umbilicate or with central depression, peach orange (6C8–6B7) to reddish-brown (“Cinnamon-Rufous”); margin incurved and entire, becoming eroded or split and sometimes wavy in age. Spines 1–8 mm long, shortest near margin, adnate to subdecurrent, white to pale orange (5A4, 6A3). Stipe 15–60 × 5–20(30) mm, central or eccentric, equal or widening to bulbous base, sometimes with up to four basidiomes fused together at base, texture smooth, white to dull tan, bruising orange-brown (6B8–7D7 or “Ochraceous-Buff”). Context fleshy, white to dull cream-brown, staining not observed. Odor mild or slightly sweet. Taste mild.

Basidiospores 8.5–*8.9*–10 μm × 7.5–*8.5*–9.5(10) μm, *Q*= 1.00–*1.05*–1.14(1.20) (n=34/3), globose to subglobose, smooth, hyaline in KOH. Basidia 48–61 × 8.5–10.5 μm with 3–4 sterigmata. Pileipellis an interwoven cutis, hyphae smooth, cylindrical, thin-walled, mostly 4–6 μm wide. Clamp connections present.

###### Distribution.

Southeastern U.S. – North Carolina (type), Tennessee, and Georgia.

###### Ecology.

In mixed woods with *Quercus*, *Pinus*, *Tsuga*, *Fagus*, *Betula*, *Carya*, *Liriodendron*. July to December.

###### Other specimens examined.

UNITED STATES. Georgia: Chatham County, Wormsloe Plantation, in duff with *Quercus*, *Pinus*, 8 m, 26 Dec 1976, J.H. Restivo JHR40605 (TENN 040605). White County, Unicoi State Park, Unicoi to Helen Trail, solitary beside trail with *Pinus* and mixed hardwoods, 460 m, 16 Jul 2017, J.E. Uehling RAS165 (TENN 073026). White County, Unicoi State Park, Unicoi to Helen Trail, solitary beside trail with *Pinus* and mixed hardwoods, 460 m, 16 Jul 2017, R.A. Swenie RAS169 (TENN 073048). Tennessee: Great Smoky Mountains National Park, under *Pinus*, *Tsuga*, *Quercus*, *Acer*, Meig’s Creek Trail, 580 m, 30 Sep 2004, E.B. Lickey TFB12311 (TENN 060359). Great Smoky Mountains National Park, Cades Cove, scattered in mixed forest with *Fagus*, *Quercus*, *Carya*, *Pinus*, 520 m, 23 Nov 2013, R.A. Walter RAW18 (TENN 073005). Great Smoky Mountains National Park, Schoolhouse Gap Trail, scattered beneath *Pinus*, *Tsuga*, *Quercus*, *Betula*, 550 m, 26 Oct 2013, K.E. Rewcastle KER016 (TENN 073006). Great Smoky Mountains National Park, trail to Look Rock fire tower, some caespitose and forming a ring among *Quercus* litter on trail, 800 m, 17 Nov 2009, E.E. Austin EEA171109-1 (073744). Great Smoky Mountains National Park, Trillium Gap Trail, 9 Jul 2017, B.P. Looney BPL931 (TENN 073028). Big Ridge State Park, Ghost House Loop Trail, scattered to caespitose under *Quercus*, *Fagus*, *Carya*, *Pinusvirginiana*, 320 m, 9 Nov 2015, R.A. Swenie RAS053 (TENN 070846).

###### Discussion.

*Hydnumsubconnatum* is known from the southeastern U.S. in a range of low elevation mixed forests such as oak-pine or hemlock-pine mixed with oak and beech. All known specimens are reported under 1000 m elevation. Basidiomes can occur in caespitose clusters with the stipe bases and two or more pilei fused together. The pileus coloration is highly variable, however, ranging from deep orange to pale peach and fading to tan towards the margin, making this a difficult species to distinguish at a glance. *Hydnumcaespitosum* Banning ex Peck (non *H.caespitosum* Valenti), described from Maryland, occurs “at roots of trees and near old stumps” and is much paler in coloration, depicted by Banning in her painting as a yellowish species. Furthermore, our examination of the holotype of *H.caespitosum* revealed that basidiospores are much smaller in that species than in *Hydnumsubconnatum*. The new name *H.geminum* is proposed below for *H.caespitosum* Banning ex Peck.

Specimens of *H.subconnatum* form a monophyletic group with support values <70% (ML) and <0.95 (BI). ITS sequence variation is relatively low (<1%) among sampled specimens of *H.subconnatum*, but the clade is highly dissimilar (8% sequence divergence) from Mexican taxa (Genbank KR135344-KR135345) that form a well-supported sister lineage.

##### Hydnum cuspidatum

Taxon classificationFungiCantharellalesHydnaceae

Swenie & Matheny sp. nov.

Figs 4B , 5M

###### Diagnosis.

Closely related to *Hydnumumbilicatum* but differs from it by ITS sequence divergence as well as more elliptic basidiospores. Known so far in the southeastern and upper midwest United States. Differs from *H.aerostatisporum* by the smaller basidiomes and slightly larger basidiospores.

###### Type.

UNITED STATES. Tennessee: Big South Fork National River & Recreation Area, John Litton Farm Trail (36.4960; -84.6700), on soil with *Quercus*, *Tsuga*, *Pinus*, 425 m, 29 Oct 2017, R.A. Swenie RAS246 (holotype: TENN 073068).

###### Etymology.

*cuspidatum* (L.), tapering to a fine, sharp point, in reference to the spines.

###### Description.

Pileus (11)15–50 mm wide, round to oval or irregular and reniform, convex when young, becoming plane or depressed, margin incurved and entire, becoming irregularly wavy or degraded; surface glabrous, sometimes floccose-scaly or scabrous near the umbilicus, dull orange to deep orange-brown (5A6–6B7–6D8, “Tawny” to “Mikado Brown”), olive-brown with KOH, at times faded in color towards the margin. Spines 1–8 mm long, shorter near the margin, adnate, pale buff, cream-orange, or tan-orange (7.5YR 8/4–8/6 or 5A3–A5). Stipe 15–50 × 3–10(12) mm, central or eccentric, equal or enlarged towards base, sometimes curved, texture smooth, buff to peach-brown (5A2–A3 to 5B6–C6), sometimes with hazy thin white patches especially towards apex, staining only very slightly light brown (10YR 7/4–7/6 or 5A–B7); cottony white basal mycelium often present. Context often hollow, flesh white to cream. Odor not distinctive or sweet and fruity. Taste not distinctive.

Basidiospores (7)7.5–*8.5*–9.5(10.0) μm × 6–*7.2*–8.5 μm, *Q*=1.01–*1.18*–1.38(1.52) (n=99/6), subglobose to irregularly rounded-elliptic, smooth, thin-walled, hyaline in KOH. Basidia 39–56 × 7–9 μm with 3–4 sterigmata. Pileipellis an interwoven cutis, hyphae smooth, cylindrical, thin-walled, mostly 4–7 μm wide. Clamp connections present.

###### Distribution.

Eastern U.S. – Michigan (KU612606, MG162266), North Carolina, Tennessee (type), and Georgia.

###### Ecology.

In deciduous or mixed woods with *Quercus*, *Pinus*, *Tsuga, Fagus, Betula, Carya, Carpinus, Picea*. June to October.

###### Other specimens examined.

UNITED STATES. Georgia: White County, Unicoi State Park, Unicoi to Helen Trail, solitary with *Pinus* and mixed hardwoods, 460 m, 16 Jul 2017, R.A. Swenie RAS167 (TENN 073046). North Carolina: Blue Ridge Parkway near Little Switzerland, deciduous woodlot with *Quercus* and *Rhododendronmaximum*, 1050 m, 19 Aug 2016, H. Hopping RAS106 (TENN 073016). McDowell County, Armstrong Creek, mixed woods, 450 m, 19 Aug 2016, J. Roberts RAS098 (TENN 073008). Great Smoky Mountains National Park, Big Creek Campground, under *Tsuga*, *Carpinus*, *Betula*, *Fagus*, *Quercus*, 525 m, 29 Jul 2017, B.P. Looney BPL989 (TENN 073033). Great Smoky Mountains National Park, Cataloochee Divide Trail, solitary with *Quercus*, *Betula*, *Tsuga*, 1525 m, 9 Sep 2017, RAS218 (TENN 073059). Great Smoky Mountains National Park, Balsam Mountain Road, 1525 m, 15 Aug 2005, E.B. Lickey TFB12725 (TENN 073033). Great Smoky Mountains National Park, Heintooga Round Bottom Road, scattered on embankment with *Betula*, *Picea*, 1525 m, 17 Aug 2017, R.A. Swenie RAS205 (TENN 073056). Tennessee: Great Smoky Mountains National Park, Tremont, Buckeye Trail, with *Tsuga*, *Betula*, 490 m, 23 Jun 2017, R.A. Swenie RAS151 (TENN 073038). Great Smoky Mountains National Park, Schoolhouse Gap Trail, scattered on embankment with *Quercus*, *Betula*, *Pinus*, 550 m, 18 Jul 2017, R.A. Swenie RAS160 (TENN 073043). Anderson County, Oak Ridge, UT Arboretum, on soil in forest with *Quercus*, *Carya*, 300 m, 26 Oct 2009, J. Heggan JRH102609-3 (TENN 071998).

###### Discussion.

*Hydnumcuspidatum* is closely related to *H.umbilicatum* and is difficult to distinguish by morphology alone. As in *H.umbilicatum*, there is high variability in basidiome stature and color. The basidiospores of *H.cuspidatum* have a slightly higher *Q* value on average (1.18) than *H.umbilicatum* (1.06), otherwise phylogenetic analysis of ITS data is needed to distinguish the two species reliably. *Hydnumcuspidatum* occurs in deciduous or mixed forests in the midwest and southeastern U.S., where it can co-occur with *H.umbilicatum*.

The ITS sequences of *H.cuspidatum* have relatively high intraspecific variation (up to 3%) compared to other North American *Hydnum* species. The lack of distinguishing morphological or ecological features deters further differentiation into separate taxa at this time.

##### Hydnum umbilicatum

Taxon classificationFungiCantharellalesHydnaceae

Peck, Ann. Rep. N.Y. St. Mus. 54: 953 (1902)

Figs 5C, D , 6N, O

###### Type.

UNITED STATES. New York: Rensselaer County, Sandlake, ground in thin woods, September, ca. 1901, C.H. Peck (holotype: NYS-F-3258). **Epitype.** UNITED STATES. New York: Cortland County, Lime Hollow Nature Center Tunison Aquatic Lab (42.5578; -76.2486), on humus in wet, boggy area with *Tsuga*, *Betulaalleghaniensis*, 27 Aug 2014, T.J. Baroni 10651TJB (CORT 012241, epitype here designated).

###### Description.

Pileus 15–70 mm wide, round, conico-campanulate to irregularly convex, disc shallowly depressed to umbilicate; surface matt, glabrous or felty-fibrillose, orange-cream to orange-brown (5C6–8); margin entire and incurved when young to undulating in age, often paler in color than the rest of the pileus. Spines 1–8 mm long, aculeate, adnexed, fleshy pinkish to light orange (5A2–3). Stipe 20–80 × 4–15 mm, central or eccentric, equal to slightly enlarged downwards, glabrous or densely matted with fluffy fibrillose; white to peachy-pallid buff, staining ochre to medium brownish orange (“Mars Yellow” to “Orange Rufous”). Context white. Odor mild or pleasant. Taste mild, sometimes with nutty aftertaste.

Basidiospores 7.5–*8.4*–9.5 μm × 7–*8*–9 μm, *Q*=1.00–*1.06*–1.18 (n=97/5), globose to subglobose, smooth, thin-walled, hyaline in KOH. Basidia 43–52 × 7.5–10 μm with (1)2–4 sterigmata. Pileipellis an interwoven cutis, hyphae smooth, cylindrical, thin-walled, mostly 4–8 μm wide. Clamp connections present.

###### Distribution.

Eastern North America – Michigan, Massachusetts, New York (type), Tennessee, North Carolina, Newfoundland and Labrador (GenBank KX388676), and Quebec (GenBank KX388675).

###### Ecology.

In coniferous or mixed woods with *Tsuga*, *Pinus*, *Abies*, *Quercus*, *Betula*, *Fagus*. July to November.

###### Other specimens examined.

UNITED STATES. Massachusetts: Worcester County, Rutland State Park, in arcs scattered singly or gregarious with *Quercus*, *Pinusstrobus*, 260 m, 1 Nov 2003, P.B. Matheny PBM2511 (TENN 066875). Michigan: Marquette County, Big Bay, Alder Creek, 240 m, 2 Sep 1971, R.H. Petersen TFB36346 (TENN 036346). New York: Tompkins County, Ridgewood Reserve, 305 m, 13 Sep 2007, O. Akinyemi OA14 (CORT 008175). Tompkins County, Eames Bog, 325 m, 22 Sep 2011, B. Demo BD14 (CORT 007319). North Carolina: Transylvania County, Pisgah National Forest, Yellow Gap Road, under *Quercus*, 850 m, 18 Jul 2000, Jason TFB9766 (TENN 058667). Blue Ridge Parkway near Little Switzerland, coniferous woodlot, 1000 m, 19 Aug 2016, N. Byers RAS103 (TENN 073013). Yancey County, Carolina Hemlocks Recreation Area, picnic area under *Tsugacarolinensis*, *Quercus*, possibly *Betula* or *Carpinus*, 840 m, 29 Sep 2017, R.A. Swenie RAS234 (TENN 058667). Yancey County, Mount Mitchell State Park, Balsam Mountain trail, under *Abiesfraseri*, 1920 m, 19 Aug 2016, R.A. Swenie RAS099 (TENN 073009). Yancey County, Mount Mitchell State Park, Balsam Mountain trail, under *Abiesfraseri*, 1920 m, 19 Aug 2016, R.A. Swenie RAS101 (TENN 073011). Yancey County, Mount Mitchell State Park, Balsam Mountain trail, under *Abiesfraseri*, 1920 m, 29 Sep 2017, R.A. Swenie RAS238 (TENN 073066). McDowell County, Armstrong Creek trail, under *Quercus*, *Pinus*, *Liriodendron*, 450 m, 30 Sept 2017, R.A. Swenie RAS239 (TENN 073067). Great Smoky Mountains National Park, Big Creek, Baxter Creek Trail, with *Tsuga*, *Pinus*, *Quercus*, 936 m, 25 Aug 2004, E.B. Lickey TFB12039 (TENN 060288). Tennessee: Great Smoky Mountains National Park, Maddron Bald Trail, scattered on soil and hardwood leaf litter with *Tsuga*, *Quercus*, *Fagus*, *Pinus*, 575 m, 29 Aug 2013, S.A. Trudell SAT1324109 (TENN 068871). Great Smoky Mountains National Park, Greenbrier, Injun Creek Trail, under *Tsuga*, 450 m, 18 Nov 2004, E.B. Lickey TFB12369 (TENN 060445). Great Smoky Mountains National Park, Cosby, Gabes Mountain Trail, with *Tsuga* and mixed hardwoods, 685 m, 16 Oct 2006, E.B. Lickey TFB13482 (TENN 061745). Big South Fork National River & Recreation Area, John Litton Farm Trail, scattered at base of dead *Tsuga*, in mixed woods with *Tsuga*, *Quercus*, *Pinus*, 425 m, 29 Oct 2017, R.A. Swenie RAS247 (TENN 073181).

###### Discussion.

*Hydnumumbilicatum* is widespread in eastern North America at low and high elevations, mostly in conifer-dominated forests or mixed woods including conifers. The macromorphology can vary dramatically among basidiomes with some specimens displaying the namesake umbilicate pileus while others do not. The presence of an umbilicus is not a unifying taxonomic feature as its presence has been observed in several distantly related clades of *Hydnum*. Peck (1901) included a color plate illustration with his description depicting basidiomes with thin, convex, umbilicate pilei and slender stipes that are slightly longer than the diameter of the pileus. Unfortunately, we were unable to obtain DNA sequences from the type collection. However, in comparison to other closely related clades, specimens of *H.umbilicatum* have slightly larger globose to subglobose basidiospores averaging 8.4 × 8 μm with average *Q* values below 1.08, which closely matches our spore measurements of the holotype. In addition, Peck mentioned that “sometimes a definite line separates the paler margin from the more highly colored center of the pileus”, a trait that has been observed in several of the specimens of this species that cluster in a single ITS lineage.

**Figure 6.**
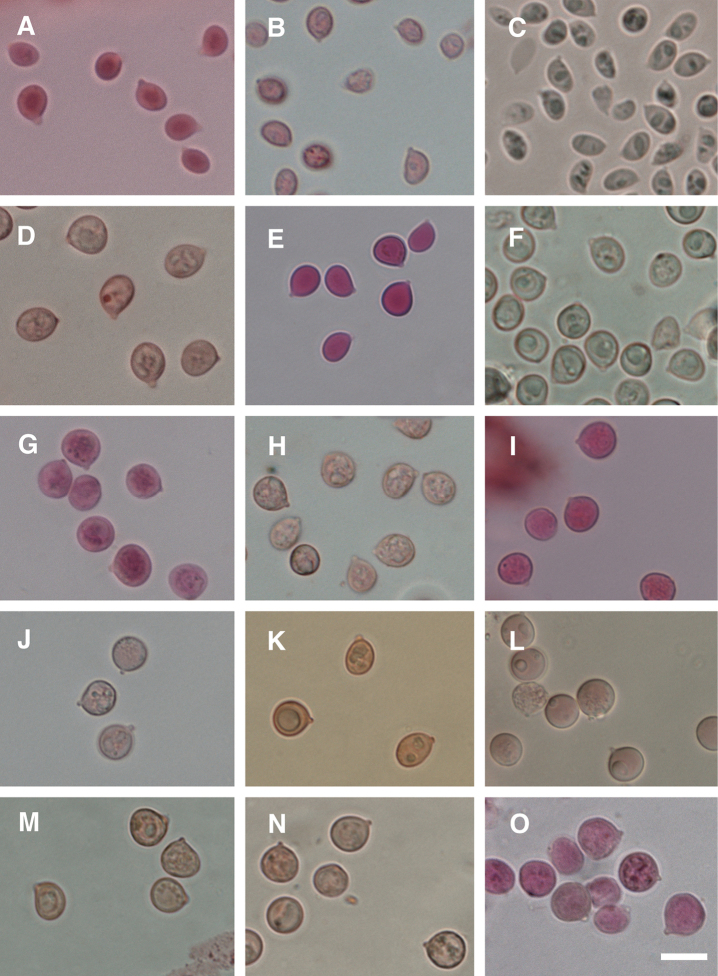
Basidiospores of *Hydnum* species. **A***H.albidum* 9623TJB (CORT 014489) **B***H.alboaurantiacum* TFB9833 (TENN 058812) **C***H.albomagnum* PBM2512 (TENN 066858) **D***H.subtilior* PBM3868 (TENN 067482) **E***H.subolympicum* AJR14 (TENN 073004) **F***H.vagabundum* 10782TJB (CORT 014461) **G***H.ferruginescens* MH16005 (TENN 073549, holotype) **H***H.aerostatisporum* MK9181403 **I***H.canadense* RAS100 (TENN 073010) **J***H.mulsicolor* MH16006 (TENN 073550) **K***H.quebecense* JG003 (CORT 007330) **L***H.subconnatum* RAS053 (TENN 070846) **M***H.cuspidatum* BPL989 (TENN 073033) **N***H.umbilicatum* 10651TJB (CORT 012241, epitype) **O***H.umbilicatum* Peck (NYS-F-3258, holotype). Scale bar: 10 μm.

#### Species from eastern North America – Incertae sedis

##### Hydnum geminum

Taxon classificationFungiCantharellalesHydnaceae

Swenie & Matheny nom. nov.

Fig. 7

≡ Hydnumcaespitosum Banning ex Peck, Rep. N.Y. St. Mus. 44: 74 (1891), non Valenti (1868)

###### Type.

UNITED STATES. Maryland: Carroll County, in clusters at the roots of trees and near old stumps, Aug-Sep, ca. 1880, M.E. Banning (holotype: NYS-F-3506).

###### Etymology.

*geminum* (L.), twin, in reference to the clustered habit

###### Description.

Pileus up to 40 mm wide, subconfluent, convex to expanded or subplane, subregular; surface appressed-fibrous, pale ochre, yellow, or dark flesh-colored. Spines short (<3 mm long), conical, acute, decurrent, pale ochre or light flesh color. Stipe up to 60 × 10 mm, united at the base, subcylindrical, subflexuous, floccose above, subglabrous below, whitish, staining yellow where bruised, solid. Context fleshy, white, turning yellow where cut. Taste mild.

Basidiospores 6–*6.4*–7 μm × 4.5–*5.2*–6 μm, *Q*=1.09–*1.24*–1.36 (n=12/1), broadly elliptic to subglobose, smooth, thin-walled, hyaline in KOH. Basidia not reviving, with 4–5 sterigmata. Pileipellis not observed. Clamp connections present.

###### Distribution.

Eastern U.S. – Maryland (type).

###### Ecology.

In clusters at the roots of trees and near old stumps, August to September.

###### Discussion.

The new binomial *H.geminum* is introduced to replace the illegitimate name *H.caespitosum* Banning ex Peck, which is a later homonym of *H.caespitosum* Valenti. The gross morphological description here is reproduced from Banker (1906) after reformatting for style, and measurements appear to be based on dried specimens. Peck’s protologue, Banning’s painting, and Banker’s notes depict a species best characterized by the overall yellowish color, short decurrent spines, flavescent or yellowing flesh, mild taste, and broadly elliptic to subglobose basidiospores that are mostly 6.5 × 5 μm in size. Although specimens of *H.subconnatum* (described above) may share the similar caespitose or clustered habit, it differs from *H.geminum* by the peach-orange to dark orange-brown pileus, longer non-decurrent spines, and a stipe that bruises orange-brown, not yellow. In addition, the basidiospores of *H.subconnatum* are larger than in *H.geminum* – 8.5–9.5 × 7.5–9 μm. Basidiomes of *H.subtilior* sometimes have an overall pale yellow tone and stain when bruised or cut in half, but basidiospores are smaller in *H.geminum*, and the overall basidiome stature of the holotype appears much stouter than in the generally slender *H.subtilior*.

Upon examining the holotype of *H.caespitosum*, we found that basidiomes had very short spines (<1 mm) with very few spores and basidia. This aligns with Peck’s notes indicating he did not obtain spores and suggests the holotype consists of immature basidiomes.

We have not yet recorded *H.geminum* in eastern North America. Banker (1906) refers to a collection made by Earle from Connecticut now housed at NCU (NCU-F-0012251). We have not re-examined this collection, but Banker describes it as somewhat darker than the type.

**Figure 7.**
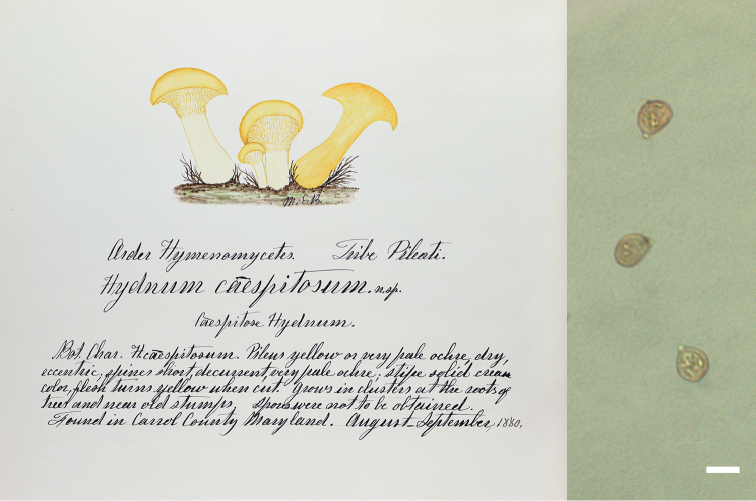
Holotype and basidiospores of *Hydnumcaespitosum*. Scale bar: 5 μm.

#### Key to species of *Hydnum* in eastern North America

Note that the five species in couplet 17 are most reliably distinguished by phylogenetic analysis of ITS sequences.

**Table:** 

1	Pileus white, cream, yellow, peach, pale orange, or light tan before handling	**2**
–	Pileus darker than above, orange to tawny brown before handling	**11**
2	Pileus mostly pale white to off-white or cream	**3**
–	Pileus mostly with tones of yellow, peach, pale orange, or light tan	**7**
3	Pileus small to medium-sized, <60 mm wide at maturity	**4**
–	Pileus larger than above, >60 mm wide at maturity	**5**
4	Basidiomes staining *bright orange* within two minutes of handling	*** H. alboaurantiacum ***
–	Basidiomes remaining white where handled or slowly staining orange-brown to ochre-brown after several minutes to hours	*** H. albidum ***
5	Pileus with adhering litter debris	*** H. albomagnum ***
–	Pileus free of adhering debris	**6**
6	Basidiomes staining bright orange where handled, spores <7 μm long	*** H. alboaurantiacum ***
–	Basidiomes not staining bright orange where handled, spores mostly >7 μm long	*** H. vagabundum ***
7	Stipe >20 mm wide	*** H. subolympicum ***
–	Stipe <20 mm wide	**8**
8	Basidiospores mostly 4–6 × 3–5 μm	*** H. alboaurantiacum ***
–	Basidiospores larger than above, mostly 6–9 × 5–7.5 μm	**9**
9	Basidiospores mostly 6–7 × 5–6 μm	*** H. geminum ***
–	Basidiospores mostly 7–9 × 5.5–7.5 μm	**10**
10	Pileus light cream yellow to cream orange buff, known only from the southeastern U.S. and Mexico in deciduous or mixed woods	*** H. subtilior ***
–	Pileus pale orange, known only from eastern Canada and the western US in coniferous or mixed woods	*** H. washingtonianum ***
11	Basidiomes caespitose	*** H. subconnatum ***
–	Basidiomes solitary or scattered	**12**
12	Spines decurrent	*** H. mulsicolor ***
–	Spines subdecurrent or adnate	**13**
13	Basidiospores mostly 5.5–7.5 × 5–7.5 μm	*** H. ferruginescens ***
–	Basidiospores larger than above, 6.5–10 × 6–9.5(10) μm	**14**
14	In *Sphagnum* in conifer-dominated woods	**15**
–	On soil in deciduous, mixed, or coniferous woods	**16**
15	Basidiospores 6.5–8 × 6–7.5 μm	***H. sp. AS30***
–	Basidiospores larger than above, 8–9.5 × 7–9 μm	*** H. quebecense ***
16	Basidiospores 8.5–10 × 7.5–9.5 μm, known only from the southeastern U.S. in mixed hardwoods under 900 m elevation	*** H. subconnatum ***
–	Basidiospores smaller than above, 7–9.5(10) × 6–9 μm, widespread or known only from northeastern North America in coniferous or mixed woods at various elevations	**17**
17	Known from mixed and hardwoods at all elevations, common and widespread in eastern U.S. (Gulf coast, southeast, midwest)	*** H. aerostatisporum ***
–	Known only from coniferous woods at low elevation in Quebec	*** H. submulsicolor ***
–	Known only from coniferous woods at low elevation in northeastern Canada and high elevation in southeastern U.S.	*** H. canadense ***
–	Known from coniferous, mixed forests, and hardwoods at various elevations in midwest and southeastern U.S.	*** H. cuspidatum ***
–	Known from coniferous and mixed woods at all elevations, common and widespread in eastern North America (southeast, northeast, midwest)	*** H. umbilicatum ***

## Supplementary Material

XML Treatment for Hydnum albidum

XML Treatment for Hydnum alboaurantiacum

XML Treatment for Hydnum albomagnum

XML Treatment for Hydnum subtilior

XML Treatment for Hydnum subolympicum

XML Treatment for Hydnum vagabundum

XML Treatment for Hydnum washingtonianum

XML Treatment for Hydnum ferruginescens

XML Treatment for Hydnum aerostatisporum

XML Treatment for Hydnum canadense

XML Treatment for Hydnum mulsicolor

XML Treatment for Hydnum quebecense

XML Treatment for Hydnum

XML Treatment for Hydnum subconnatum

XML Treatment for Hydnum cuspidatum

XML Treatment for Hydnum umbilicatum

XML Treatment for Hydnum geminum

## Supplementary materials

Supplementary material 1 Specimen data for sequences produced in this study Data type: specimen data This is an open access article distributed under the terms of the Creative Commons Attribution License (CC BY 4.0), which permits unrestricted use, distribution, and reproduction in any medium, provided the original author and source are credited.Rachel A. Swenie, Timothy J. Baroni, P. Brandon Matheny

Supplementary material 2 BI phylogeny of global species of *Hydnum* shown as a rectangular tree Data type: phylogeny data Explanation note: Posterior probabilities are shown above branches. This is an open access article distributed under the terms of the Creative Commons Attribution License (CC BY 4.0), which permits unrestricted use, distribution, and reproduction in any medium, provided the original author and source are credited.Rachel A. Swenie, Timothy J. Baroni, P. Brandon Matheny
